# Structural basis for the targeting of complement anaphylatoxin C5a using a mixed L-RNA/L-DNA aptamer

**DOI:** 10.1038/ncomms7481

**Published:** 2015-04-22

**Authors:** Laure Yatime, Christian Maasch, Kai Hoehlig, Sven Klussmann, Gregers R. Andersen, Axel Vater

**Affiliations:** 1 Department of Molecular Biology and Genetics, Aarhus University, Gustav Wieds Vej 10C, DK-8000 Aarhus, Denmark; 2 NOXXON Pharma AG, Max-Dohrn-Strasse 8-10, 10589 Berlin, Germany

## Abstract

L-Oligonucleotide aptamers (Spiegelmers) consist of non-natural L-configured nucleotides and are of particular therapeutic interest due to their high resistance to plasma nucleases. The anaphylatoxin C5a, a potent inflammatory mediator generated during complement activation that has been implicated with organ damage, can be efficiently targeted by Spiegelmers. Here, we present the first crystallographic structures of an active Spiegelmer, NOX-D20, bound to its physiological targets, mouse C5a and C5a-desArg. The structures reveal a complex 3D architecture for the L-aptamer that wraps around C5a, including an intramolecular G-quadruplex stabilized by a central Ca^2+^ ion. Functional validation of the observed L-aptamer:C5a binding mode through mutational studies also rationalizes the specificity of NOX-D20 for mouse and human C5a against macaque and rat C5a. Finally, our structural model provides the molecular basis for the Spiegelmer affinity improvement through positional L-ribonucleotide to L-deoxyribonucleotide exchanges and for its inhibition of the C5a:C5aR interaction.

## Abstract (web summary)

Spiegelmers are mirror-image oligonucleotide aptamers designed for therapeutic use. Here the authors describe the crystal structure of the mixed L-RNA/L-DNA Spiegelmer NOX-D20 bound to complement component C5a, a key mediator of the innate immune response and clinical target in acute and chronic inflammatory disorders.

## Graphical abstract

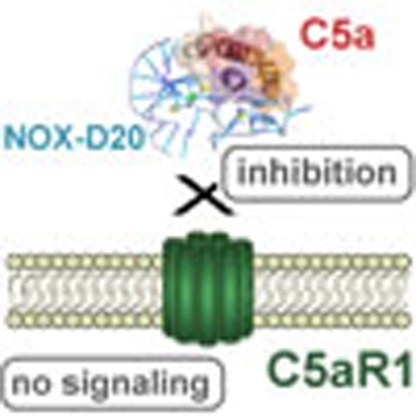

Complement is a central component of innate immunity that provides a first line of defence against invading pathogens and participates in immune surveillance^1^,^2^,^3^. It also bridges the innate and adaptive immunity, initiates the inflammatory response and helps maintaining homeostasis^2^,^3^,^4^,^5^. Detection of foreign microorganisms or damaged host cells triggers complement activation, leading to opsonization and assembly of the lytic membrane attack complex (MAC)^1^,^2^. In addition, the anaphylatoxins C3a and C5a are released and thereafter function as signalling molecules through their cognate G-protein-coupled receptors present on the host cells^6^,^7^,^8^. By targeting its receptors C5aR1 and C5aR2 on a wide range of immune cells^6^,^9^ as well as endothelial cells^10^, C5a promotes inflammation, vascular permeability and coagulation^6^,^7^,^8^,^9^,^11^. Although complement activation and inflammation are essential for the host defence and the healing process following tissue damage and infection, unduly elevated levels of C5a may promote and/or exacerbate pathological conditions underlying various acute and chronic inflammatory disorders such as acute lung injury, ischaemia-reperfusion injuries, sepsis, transplant rejection, rheumatoid arthritis, allergy and asthma^9^,^11^,^12^,^13^,^14^.

Over the last decades, intensive efforts have been made to design potent inhibitors applicable for the treatment of complement-mediated diseases^15^,^16^,^17^. In particular, specific targeting of C5a and its receptors allows for tuning down anaphylatoxin-mediated inflammation while maintaining opsonization and MAC-mediated bacteriolysis^18^,^19^. This strategy is likely to be of particular value in patients at increased risk of infection such as critically ill and immunosuppressed patients. In this line of efforts, a series of plasma-stable C5a-inhibiting aptamers composed of non-natural, mirror-image L-nucleotides has been generated. Mirror-image aptamers, also referred to as Spiegelmers (from German *Spiegel*=*mirror*), possess the high target specificity of conventional aptamers and are in addition plasma nuclease resistant, therefore having high therapeutic and diagnostic potential^20^. Spiegelmers are generated by the SELEX process (Systematic Evolution of Ligands by EXponential enrichment)^21^ from oligonucleotide libraries in the natural D-configuration but with the addition of two chiral inversion steps. In the first step, the enantiomer of the target molecule is synthesized (here murine D-C5a). Next, aptamers from a D-oligoribonucleotide library with 10^15^ different sequences are identified using SELEX and ranked for binding to the selection target. In a second chiral inversion step, the best aptamer sequences are truncated to the minimal size without loss of affinity and are then synthesized in their mirror-image L-configuration, thereby resulting in Spiegelmers binding to the natural murine L-C5a (Fig. 1a). Finally, through post-SELEX optimization, the initial C5a-binding, 44 nucleotides-long, L-RNA Spiegelmer NOX-D19 was modified by six positional ribo-to-deoxyribonucleotide exchanges, leading not only to improved affinity but also allowing a further truncation by four nucleotides, thus yielding the 40 nucleotide-long Spiegelmer NOX-D20 (ref. 22). This L-aptamer binds both human and murine C5a with picomolar affinities and has shown efficacy in a rodent model of polymicrobial sepsis induced by cecal ligation and puncture^22^. NOX-D20 is currently under consideration for preclinical and clinical development.

Here we report the crystal structures of NOX-D20 in complex with either murine C5a (mC5a) or its C-terminally desarginylated version mC5a-desArg, at 1.8 and 2.0 Å resolution, respectively. The structures reveal a complex 3D architecture for the Spiegelmer, including a left-turning double-helix and an intramolecular G-quadruplex stabilized by a Ca^2+^ ion. These features allow the L-aptamer to wrap around the C5a molecule, forming a complex with tight shape complementarity. The NOX-D20:C5a binding mode observed in the structure is further validated by mutational studies using surface plasmon resonance (SPR) and allows to explain NOX-D20 specificity for human and mouse C5a species as opposed to macaque and rat C5a^22^, as well as the increased affinity obtained following ribo-to-deoxyribonucleotide exchange. Finally, these data provide a molecular basis for the inhibitory properties of NOX-D20 towards the C5a:C5aR interaction.

## Results

### Structure of the NOX-D20:mC5a/mC5a-desArg complexes

The NOX-D20:C5a complexes were formed by mixing the Spiegelmer with murine recombinant C5a/C5a-desArg^23^ in a 1:1 molar ratio, in the presence of monovalent (K^+^ and Na^+^) and bivalent (Ca^2+^ and Mg^2+^) cations^22^. Crystals for the NOX-D20:mC5a and NOX-D20:mC5a-desArg complexes both displayed a P2_1_2_1_2 symmetry and diffracted to a maximal resolution of 1.8 and 2.0 Å, respectively (Table 1). The structure of the NOX-D20:mC5a complex was determined by single-wavelength anomalous diffraction (SAD) phasing using crystals derivatized with Os(NH_3_)_6_ (Table 1 and Supplementary Figs 1 and 2). The resulting model was then used to solve the NOX-D20:mC5a-desArg structure by molecular replacement (MR). Unexpectedly, the asymmetric units of both NOX-D20:mC5a and NOX-D20:mC5a-desArg crystals contained three molecules of mC5a and only two molecules of NOX-D20 (Supplementary Fig. 3a). One molecule of mC5a (red molecule in Supplementary Fig. 3a) has, however, only very few contacts with the Spiegelmer molecules and its role seems to be restricted to crystal packing stabilization. The two other mC5a molecules (beige and purple molecules in Supplementary Fig. 3a) each interact with one Spiegelmer molecule in a comparable manner. The root-mean-square deviation (r.m.s.d.) on all atoms between the two complexes present in the asymmetric unit (complexes A and B in Supplementary Fig. 3a) is 1.29 Å. The major difference between these two complexes is a repositioning of the loop connecting helices H2 and H3 in mC5a (residues Arg708 to Glu713; Supplementary Fig. 3b), which results from the rotation of the uracil base of dU30 by approximately 65° towards the neighbouring mC5a molecule, forcing the side chains of Val709, Asn710, Phe711 and Tyr712 to reorient (Supplementary Fig. 3c). Apart from this region, the two complexes are almost completely equivalent. As a more extended model could be traced for mC5a in complex A, we will describe this in the following section.

**Figure 1:**
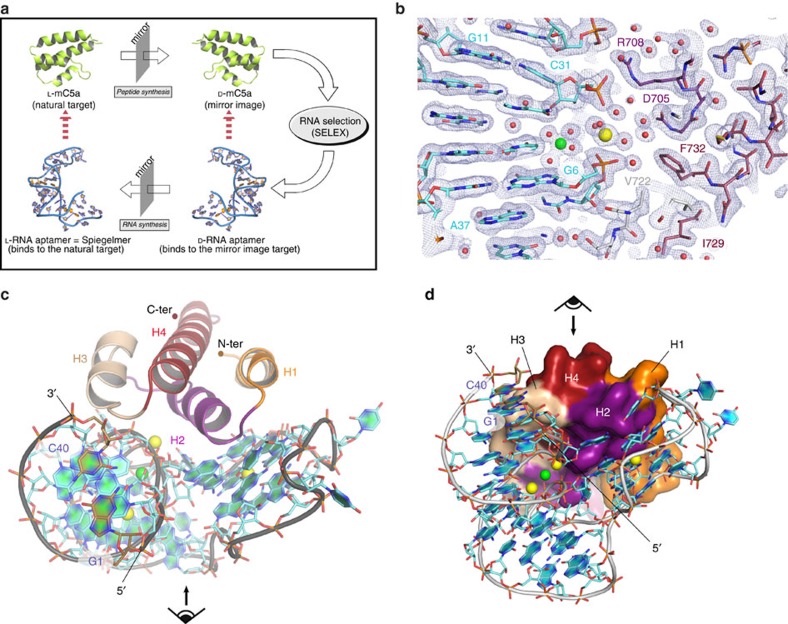
The NOX-D20:mC5a complex. (**a**) Schematic representation of the selection principle to generate mC5a-binding Spiegelmers. (**b**) Final electron density maps and final model centered on the NOX-D20:mC5a interface. The 2mF_o_-DF_c_ map is shown as grey mesh and contoured at 1*σ*. Red spheres indicate water molecules. (**c**) Structure of the NOX-D20:mC5a complex at 1.8 Å resolution. The four mC5a helices (H1–H4) are highlighted in distinct colours. The Spiegelmer is represented in grey and cyan and the 5′- and 3′-terminal bases are shown in brown. Divalent cations are indicated as spheres (yellow for Ca^2+^ and green for Mg^2+^). The corresponding direction of view in **d** is indicated by an eye and arrow. (**d**) Same as in **c** but the view is rotated by 90° and mC5a is shown as surface representation to illustrate the strong shape complementarity with the L-aptamer fold. The corresponding direction of view in **c** is indicated by an eye and arrow.

The NOX-D20:mC5a and NOX-D20:mC5a-desArg complexes are represented in Fig. 1 and Supplementary Fig. 3d, respectively. The final models encompass residues Asn679 to Pro750 for mC5a, Asn679 to Lys744 for mC5a-desArg and all 40 nucleotides for the Spiegelmer. The two structures are equivalent, with an r.m.s.d. on all atoms of 0.25 Å between the two complexes, in agreement with the fact that NOX-D20 binds both anaphylatoxins equally well^22^. mC5a adopts the canonical four-helix bundle conformation which superimposes well with the structure of isolated mC5a (PDB 4P3A^23^) with an r.m.s.d. on C-alpha atoms of 0.43 Å. The mC5a C-terminus extends away from the four-helix bundle core in the prolongation of helix H4 (Fig. 1c), reinforcing the idea that this region is highly flexible. NOX-D20 folds into a compact V-shape with the concave face of the V wrapping around the C5a molecule along the H1-H2-H3 side (Fig. 1c,d). The two binding partners display a strong shape complementarity (Fig. 1d), resulting in a total surface area buried at the NOX-D20:mC5a interface of 1,791 Å^2^, as estimated with AREAIMOL^24^.

### The anti-C5a Spiegelmer adopts a complex 3D architecture

**Figure 2:**
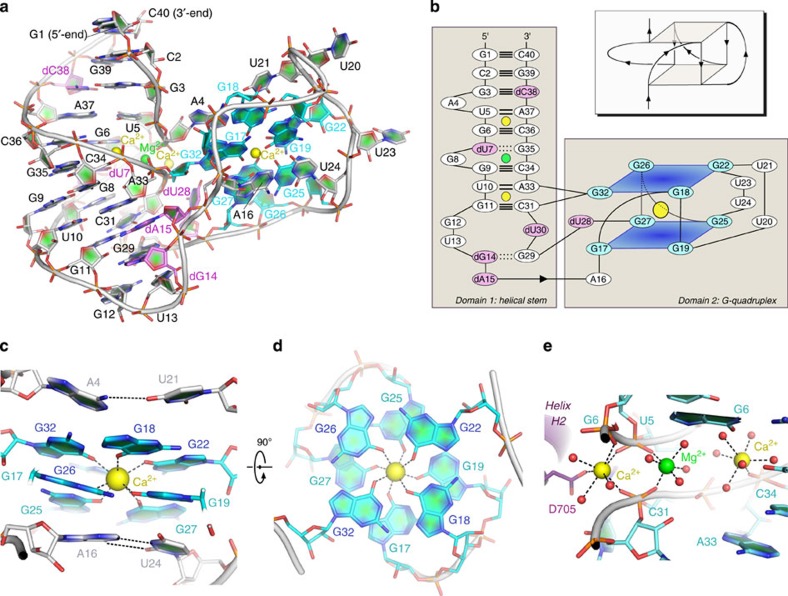
NOX-D20 adopts a complex 3D architecture. (**a**) Overview of the NOX-D20 structural organization. The deoxyribonucleotides are indicated in purple and the G-nucleotides forming the G-quadruplex are highlighted in cyan. (**b**) Secondary structure representation of the NOX-D20 Spiegelmer reflecting the presence of two strongly interconnected structural domains. The ribose-phosphate backbone is indicated by a single plain line. Watson–Crick and non-Watson–Crick base pairs in the stem domain are indicated by double (A-U) or triple (G-C) plain and double dotted lines, respectively. The topology of the G-quadruplex is indicated in the insert above the scheme. (**c**) Zoom-in on the G-quadruplex domain showing a lateral view of the two G-tetrads and the additional stacking interactions that stabilize their packing on both sides of the quadruplex. (**d**) Same as in **c** but viewed from the top of the G-quadruplex. The stabilizing Ca^2+^ ion lies in the centre of the ion channel in an almost perfect square antiprismatic coordination geometry. (**e**) Zoom-in on the minor groove of the helical stem domain, which is stabilized by three divalent cations arranged along a linear path.

NOX-D20 adopts a complex three-dimensional (3D) organization in its active, target-bound form (Figs 1 and 2). The position of the six deoxyribonucleotides (dU7, dG14, dA15, dU28, dU30 and dC38) could be unambiguously assigned in the electron density maps and almost all nucleosides are in the *anti* N-glycosidic conformation with the exception of G8, dG14, G17 and G25, which are in *syn*. Furthermore, the solvent-exposed U23 alternates between the *anti* and *syn* conformations in the two molecules of NOX-D20 contained in the asymmetric unit. The majority of the riboses adopt the energetically favourable 3′-endo and 2′-endo conformations except dU7 (4′-exo), G9 (2′-exo), dG14 (4′-exo), G25 (4′-exo) and G26 (2′-exo).

The Spiegelmer molecule can be subdivided into two distinct structural domains (Fig. 2a,b), which are strongly interconnected, thereby preserving the NOX-D20 overall architecture and maintaining the integrity of the mC5a-binding pocket. The first domain, corresponding to the first leg of the V-shape, is built around a double-stranded helical stem that connects the first third of the molecule (nucleotides 1–15) to the last third (nucleotides 29–40) in an antiparallel manner. As expected, due to the L-configuration of all the riboses, this double-helix is left-handed. The domain is shaped by an extensive network of base pairing interactions between the two strands, including eight Watson–Crick base pairs, two non-Watson–Crick base pairs (dU7-G35 and dG14-G29, dG14 being in the *syn* N-glycosidic conformation to permit the interaction), and a non-canonical interaction between the Watson–Crick face of dA15 and the ribose face of G11. Finally, G8, G11, G12, U13 and dA15 help maintaining the domain overall fold through hydrogen bonds and stacking interactions.

The second domain, that is, the second leg of the V-shape, corresponds to a large loop encompassing nucleotides 16–28 and is articulated around an intramolecular G-quadruplex consisting of two G-tetrads of same polarity that stack in a ‘partial 5/6 ring' geometry^25^. The bases of the two G-tetrads are twisted around a common perpendicular axis and the G-quadruplex is stabilized by a central Ca^2+^ ion (see later) coordinated by the O6 atoms from all eight guanosine bases (Fig. 2c,d). The packing of the two G-tetrads is further maintained by π-stacking interactions with A16 and U24 on one side of the G-quadruplex, and with A4 and U21 on the other side (Fig. 2c). These nucleotides are themselves held in position by either Watson–crick base pairing (A16-U24) or by single hydrogen bonding (A4-U21). The first G-tetrad is formed by nucleotides G17, G19, G25 and G27, whereas the second G-tetrad encompasses G18, G22, G26 and G32 (Fig. 2b–d). To allow for classical pairing of the guanine bases on both the Watson–Crick and the Hoogsteen faces, G17 and G25 from the first tetrad are in the *syn*-glycosidic conformation. The topology of the NOX-D20 G-quadruplex, with a mixed parallel–antiparallel strand orientation, two connecting loops and two connecting phosphodiester bonds, is quite unusual, in particular due to the fact that one of the G-nucleotides, G32, is provided by a distant and separate structural domain (Fig. 2b). To our knowledge, substitution of a single G from a distant site on the primary sequence has never been encountered before in G-quadruplex structures.

### The NOX-D20 fold is stabilized by divalent cations

**Figure 3:**
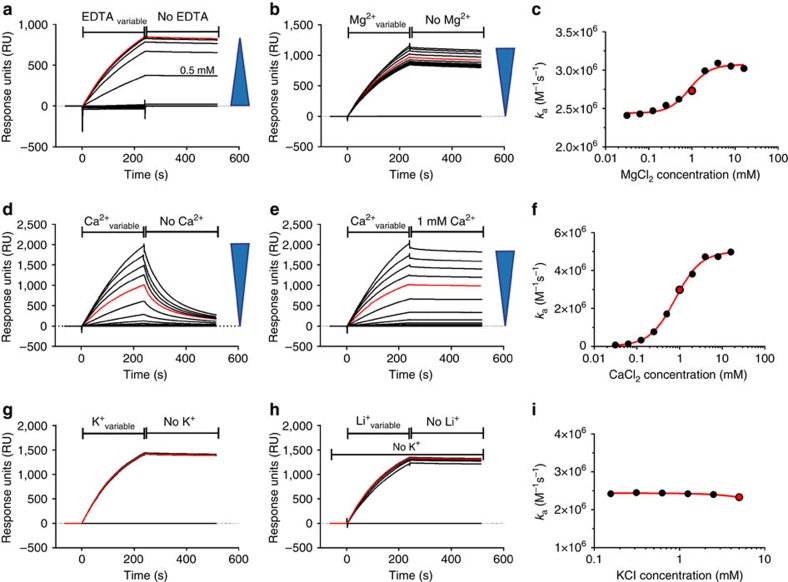
Influence of monovalent and divalent cations on mC5a recognition by NOX-D20 measured by SPR. The blue arrows indicate increasing concentrations and the red line/dot corresponds to the measurement under physiological conditions (0 mM EDTA/1 mM CaCl_2_/1 mM MgCl_2_/5 mM KCl/5 mM LiCl). (**a**) Titration of EDTA. (**b**) Titration of MgCl_2._ (**c**) Plot of the MgCl_2_ effect on the association rate constant *k*_a_. (**d**) Titration of CaCl_2_. (**e**) Titration of CaCl_2_ during the association phase and 1 mM CaCl_2_ during the dissociation phase. (**f**) Plot of the CaCl_2_ effect on the association rate constant *k*_a_. (**g**) Titration of KCl. (**h**) Titration of LiCl in the absence of KCl. (**i**) Plot of the KCl effect on the association rate constant *k*_a_.

Divalent cations are important stabilizers of RNA folds^26^. The *in vitro* selection of NOX-D20 was performed in the presence of physiological concentrations of MgCl_2_/CaCl_2_, and the NOX-D20:C5a complexes were also crystallized in a buffer containing Mg^2+^ and Ca^2+^. To assess the influence of these ions on the NOX-D20:C5a interaction, binding of NOX-D20 to mC5a was followed by SPR measurements in the presence of increasing concentrations of EDTA (Fig. 3a). EDTA inhibited the NOX-D20:mC5a interaction in a dose-dependent manner, with a complete loss of binding at concentrations above 2 mM. Furthermore, titration of MgCl_2_ or CaCl_2_ induced a dose-dependent increase in the association rate constant *k*_a_ of NOX-D20 binding to mC5a (Fig. 3b–f). The addition of either CaCl_2_ or MgCl_2_ above the physiological concentration of 1 mM further increased target binding by NOX-D20. Ca^2+^ showed a more pronounced effect, suggesting a much stronger contribution to C5a binding, whereas Mg^2+^ only showed a minor influence on *k*_a_ (Fig. 3c,f). Furthermore, Ca^2+^ is essential for the stability of the NOX-D20:mC5a complex. When Ca^2+^ is removed from the buffer, the NOX-D20:mC5a complex quickly dissociates (Fig. 3d). In contrast, a stable complex is maintained when a physiological calcium concentration (1 mM) is present during the dissociation phase, even if Ca^2+^ was lower during the association phase (Fig. 3e).

In agreement with these findings, the structure of the NOX-D20:mC5a complex revealed the presence of four ions in each Spiegelmer molecule, including one ion at the centre of the G-quadruplex (Fig. 2d,e). Anomalous difference maps calculated from diffraction data collected at high wavelength (*λ*=2.498 Å) revealed the presence of three strong peaks in each Spiegelmer molecule (Supplementary Fig. 4a). Both Ca^2+^ and K^+^ display comparable anomalous signal at this wavelength and could therefore be candidate for these positions. To identify the precise nature of the four ions, and knowing that G-quadruplexes tend to favour monovalent cations^27^, Rb^+^ or Mn^2+^ were introduced in the NOX-D20:mC5a crystallization buffer as congeners for K^+^ and Mg^2+^, respectively, that give a strong anomalous signal at specific wavelengths. No crystals could be obtained in the presence of MnCl_2_, suggesting that Mg^2+^ plays a role in NOX-D20 stabilization and/or interaction with mC5a that cannot be fulfilled by Mn^2+^. Anomalous data sets for Rb^+^-derivatized crystals in the presence of Ca^2+^ were collected at *λ*=0.81 Å, but the resulting anomalous difference maps did not reveal the presence of Rb^+^ (Supplementary Fig. 4b), suggesting that either K^+^ does not play a role in NOX-D20 folding or that in the absence of K^+^, another ion is stabilizing the Spiegelmer, in particular the G-quadruplex. Furthermore, NOX-D20 binds its target equally well in the absence of K^+^, even when Li^+^ is added (Fig. 3g–i). Li^+^ is known to alter G-quadruplex structures when replacing K^+^ (refs 28, 29). These data add another line of evidence that K^+^ does not play a role in NOX-D20 folding. Analysis of the ion–oxygen distances observed for these three ions in the final model revealed values ranging from 2.43 to 2.52 Å (Supplementary Fig. 4e), in agreement with values expected for Ca–O distances (2.43±0.11 Å (ref. 30)) and significantly lower than the values expected for K–O distances (2.81±0.10 Å (ref. 30)). To ensure that these distances were not model-biased by the presence of calcium, the Ca^2+^ ions were removed and refinement with simulated annealing was performed to remove model bias. The distances calculated between the coordinating oxygens and the centres of the peaks obtained in the resulting mF_o_-DF_c_ map were again much closer to the expected Ca–O distances than the K–O ones (Supplementary Fig. 4e). Finally, adding back either Ca^2+^ or K^+^ at these positions and performing refinement with tight restraints on the Ca–O or K–O distances led to a model superimposable to the final one when Ca^2+^ was present, whereas in the presence of K^+^, the coordinating oxygen ligands (from water molecules, from the RNA phosphate backbone or from the guanine bases of the G-quadruplex) were pushed away to the periphery of the electron density to match more closely the ideal K–O distance values implemented through the restraints, which are also observed in G-quadruplexes stabilized by potassium (Supplementary Fig. 4c,d). Taken together, these observations allowed us to unambiguously assign the three ions present in NOX-D20 structure giving strong anomalous signal at high wavelength as Ca^2+^. As SPR data suggested a slightly enhancing effect of Mg^2+^ on NOX-D20:mC5a binding and as all the other possible ions were ruled out, the last ion present in the structure was assigned as Mg^2+^, which also agrees with the hexacoordination and the average ion-oxygen distance of 2.08±0.06 Å observed for this ion in our structure (Fig. 2e).

Three of the four ions present in NOX-D20 structure align along a straight line in the middle of the helical stem in the first domain (Fig. 2a,e). Their position right within the minor groove of the double-helix leads to the tightening of the packing between the two strands, thereby bringing them to a distance of 4.5 Å in the narrowest part, corresponding to half the width of the minor groove in standard A-form RNA. The central Mg^2+^ is in an almost perfect octahedral coordination sphere formed by four water molecules and two phosphate oxygens from U5 and C31. The two Ca^2+^ surrounding the Mg^2+^ ion are heptacoordinated by water molecules, oxygens from the phosphate groups of U5, G6, C31 and C34 and, for the most peripheral calcium ion, by the side chain of Asp705 protruding from mC5a helix H2 (Fig. 2e). Finally, our anomalous difference maps conclusively show that the last Ca^2+^ is located in the second structural module of NOX-D20, at the centre of the G-quadruplex, and is coordinated by all eight guanine bases (Fig. 2c,d).

### A complex built on strong shape complementarity

**Figure 4:**
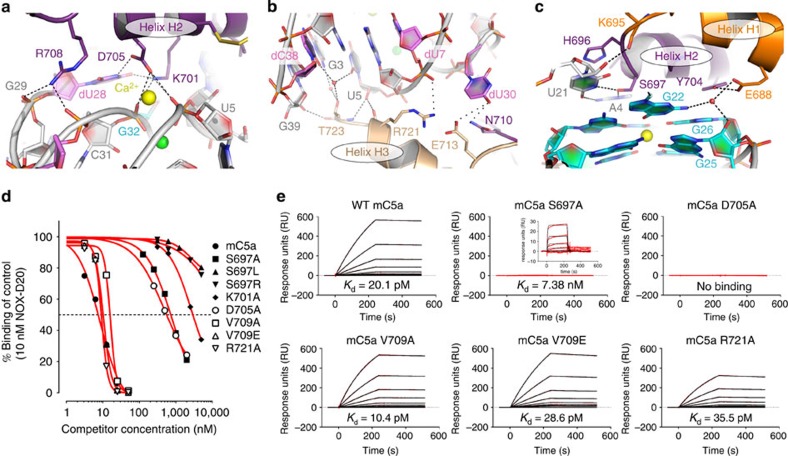
The NOX-D20:mC5a interface. (**a**) Zoom-in on the detailed interactions of mC5a helix H2 with NOX-D20 at the bottom of the Spiegelmer's binding cavity. (**b**) Detailed interactions between mC5a helix H3 and the Spiegelmer stem domain. (**c**) Detailed interactions between mC5a helices H1–H2 and the Spiegelmer G-quadruplex. (**d**) NOX-D20 binding of mC5a WT and mutants analysed by competitive SPR measurement with immobilized mC5a on the sensor chip surface, a fixed NOX-D20 concentration and increasing competitor concentrations. (**e**) Direct SPR measurement of the binding affinities of NOX-D20 for relevant mC5a mutants compared with WT mC5a using immobilized mC5a and increasing NOX-D20 concentrations. The weak interaction with mC5a S697A is shown in the magnified inset.

Within the complex, mC5a inserts into the V-shaped groove formed at the interface between the two structural domains of NOX-D20 and the Spiegelmer's binding pocket almost perfectly complements the surface shape of mC5a helices H1, H2 and H3 (Fig. 1c,d). H2 runs along the bottom of the binding cavity and forms the most extended network of interactions with the aptamer molecule at the junction between NOX-D20 domains. On the sides of the cavity, mC5a helix H1 interacts with the core region of the G-quadruplex domain, whereas helix H3 is recognized by the helical stem domain. The presence of mC5a therefore locks NOX-D20 into a rigid conformation by tightening the packing between its two structural domains. Contacts between NOX-D20 and mC5a H2 are mainly mediated by polar residues (Fig. 4a). Lys701 and Asp705, located in the middle of H2, form hydrogen bonds with U5 and G32 phosphate groups, along the minor groove of the NOX-D20 stem domain, and with the base of dU28. At the C-terminus of H2, Arg708 makes electrostatic interactions with the phosphate groups of G29 and C31. The H2-H3 loop and helix H3 further interact with the NOX-D20 helical stem domain, through long-range interactions between the side chains of Asn710 and Glu713 and the O4 atom of dU30, as well as between Arg721 and the dU7 phosphate group (Fig. 4b). These interactions are, however, loosened in the other complex of the asymmetric unit because of the alternative conformation of dU30 (Supplementary Fig. 3c). The main-chain carbonyl of Arg721 also directly engages the 2′-OH of U5 ribose in a hydrogen bond. At the end of H3, Thr723 connects the 2′-OH ribose groups from U5 and G39, and makes water-mediated hydrogen bonds with the bases from G3, U5 and dC38. On the other side of H2, residues from H1 and the H1–H2 loop make direct contacts with the G-quadruplex domain, with His696 and Tyr704 making stabilizing stacking interactions with U21 and G26, respectively, whereas the main-chain carbonyl of Lys695 forms a hydrogen bond with the 2′-OH of U21 ribose (Fig. 4c). In addition, Glu688 engages in water-mediated hydrogen bonds with G22 and G25 and holds in place Tyr704. Finally, Ser697, at the tip of the H1-H2 loop, inserts its side chain between A4 and U21 and bridges the two bases through hydrogen bonding, thereby creating a closed surface parallel to the second G-tetrad.

**Figure 5:**
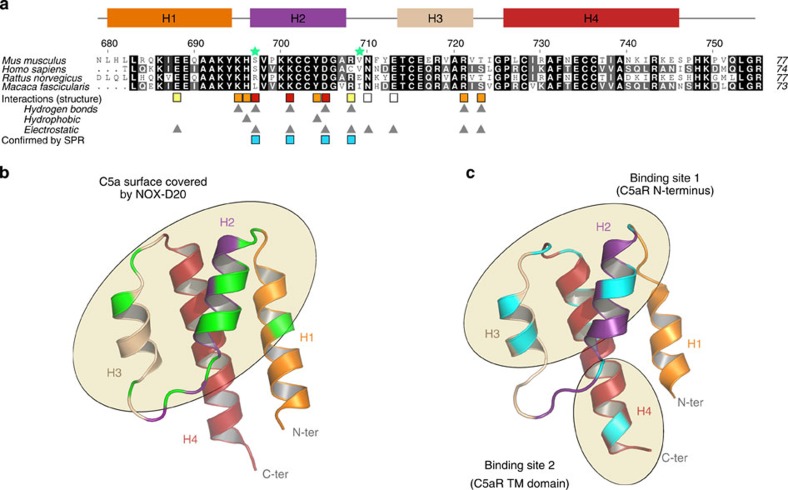
C5a alignment showing conserved residues involved in NOX-D20 binding and comparison of NOX-D20 and C5aR footprints onto C5a. (**a**) Sequence alignment for murine, human, rat and macaque C5a proteins. The secondary structure elements in mC5a are indicated above the alignment. Residues that are potentially involved in NOX-D20 selectivity towards human and murine C5a are indicated by green stars. C5a residues involved in NOX-D20 binding are marked with coloured squares below the alignment, with a gradient scale from red (strong) to white (weak) to indicate the strength of the interaction. The types of interactions involved for each residue are summarized below with grey triangles. Residues for which the interaction was confirmed by SPR are indicated with blue squares. (**b**) Footprint of NOX-D20 on mC5a based on the NOX-D20:mC5a structure. The mC5a residues directly involved in NOX-D20 binding are highlighted in green and the surface of mC5a covered by the Spiegelmer is indicated by an ellipse. (**c**) Putative footprint of C5aR onto hC5a (four-helix bundle model derived from the hC5a moiety of intact C5 (ref. 60)). The hC5a residues possibly involved in C5aR recognition are forming two distinct binding sites: positively charged residues from the C5a core presumably interact with the C5aR N-terminus, whereas the C5a C-terminus would insert in the receptor transmembrane domain^32^,^33^,^34^,^35^,^36^,^37^.

To confirm the NOX-D20:mC5a binding mode observed in the structure (Fig. 5a for summary), mutants of mC5a were generated and tested for their ability to compete the binding of NOX-D20 to immobilized mC5a using SPR measurements (Supplementary Fig. 5a). Mutants Ser697Leu, Ser697Arg and Lys701Ala showed the weakest competition with wild-type (WT) mC5a for binding to NOX-D20 (Fig. 4d), suggesting that Ser697 and Lys701 are key residues for the Spiegelmer:anaphylatoxin interaction. In addition, mutants Ser697Ala and Asp705Ala showed substantially reduced competition, whereas the competition of mutants Val709Ala, Val709Glu and Arg721Ala was comparable to that of WT mC5a. Detailed measurement of the binding affinity of NOX-D20 for these mutants revealed that the Ser697Ala mutant could still bind but with a 400- to 1,000-fold lower affinity, whereas no binding could be detected for the Asp705Ala mutant (Fig. 4e). In contrast, replacement of Arg721 by an alanine had only a minor effect on NOX-D20 binding despite its apparent contacts with the dU7 phosphate. Such replacement would, however, not disrupt the main-chain carbonyl Arg721:U5 interaction. All the mC5a mutants could efficiently induce chemotaxis of hC5aR1-expressing BA/F3 cells, suggesting that their defect in NOX-D20 binding was not due to improper folding (Supplementary Fig. 5b). The Asp705Ala mutant had, however, slightly lower chemotactic activity. In any case, mC5a mutational analysis confirmed the binding mode observed in the structure.

### A rationale for species selectivity and C5a inhibition

A similar binding mode to NOX-D20 is expected for human and murine anaphylatoxins, with a slightly higher affinity of NOX-D20 for mC5a^22^. Indeed, as shown in a previous study, characterization of NOX-D20-binding properties using commercial recombinant proteins displayed an almost tenfold higher affinity for mC5a compared with hC5a^22^. Here, we used a recombinant hC5a protein bearing an engineered Cys704Arg mutation to avoid nonspecific disulfide crosslinking via the free cysteine^23^. Surprisingly, this mutation resulted in a tenfold increase in the affinity of NOX-D20 for hC5a, as determined by SPR, yielding a *K*_d_ value comparable to that for mC5a (Supplementary Fig. 6a). Furthermore, the potency of NOX-D20 to inhibit the hC5a-Cys704Arg-induced chemotaxis of BA/F3 cells increased (Supplementary Fig. 6b). In agreement with our structure, these functional data therefore suggest that Arg708 in mC5a (hC5a Arg704) does not only contribute to the binding but is responsible for the increased affinity of NOX-D20 towards the murine anaphylatoxin as compared with WT hC5a.

To further confirm that mC5a and hC5a have a similar binding mode to NOX-D20, mutational analysis of our hC5a Cys704Arg recombinant protein was undertaken. The hC5a mutants Ser693Ala and Asp701Ala showed a 150- to 300-fold reduction in their affinity for the Spiegelmer and mutants Ser693Leu and Lys697Ala showed complete loss of binding to NOX-D20 (Supplementary Fig. 6c). Again, all hC5a mutants could efficiently trigger chemotaxis in BA/F3 cells, suggesting that their loss of activity towards NOX-D20 is solely mediated by a binding defect (Supplementary Fig. 6d). Interestingly, the Asp701Ala mutant showed full chemotactic activity, whereas the activity of its murine counterpart Asp705Ala was impaired. This residue therefore potentially contributes to the differential C5aR activation properties observed for human and murine C5a anaphylatoxins^23^. Nevertheless, our SPR data show that interaction with NOX-D20 involves the same residues on hC5a and mC5a.

NOX-D20 has a strong affinity for hC5a and mC5a but no reactivity towards rat or macaque C5a^22^. Sequence comparison between these different species highlighted two residues potentially involved in the Spiegelmer selectivity, Ser697 and Val709 (mC5a numbering), which are conserved in human and murine C5a but differ in other species^22^ (Fig. 5a). In accordance with the determined binding mode of NOX-D20 to mC5a/hC5a, our mutational analysis revealed that Ser697 (hC5a Ser693) indeed plays a crucial role in NOX-D20 binding, which is in agreement with its position on top of the G-quadruplex domain where it holds the stacked layers together (Fig. 4c). Introduction of a hydrophobic residue (such as a leucine in monkey C5a) or a large polar residue (such as an arginine in rat C5a) would break the stabilizing interaction between nucleotides A4 and U21, on the upper side of the G-quadruplex, and pull away the entire domain, thus strongly destabilizing the NOX-D20:C5a interface. In contrast to Ser697, none of the Val709 mutants (hC5a Val705) showed reduced affinity for NOX-D20, suggesting that this residue is not important for the Spiegelmer recognition (Fig. 4d,e, Supplementary Fig. 6). Indeed, Val709 is located in the H2-H3 loop and points towards the solvent on the opposite side of mC5a as compared with the NOX-D20:mC5a interface. Thus, Ser697 is solely responsible for NOX-D20 selectivity towards human and murine C5a.

Our structural and biochemical data also suggest that hC5a and hC5a-desArg most likely bind NOX-D20 as four-helix bundles, although hC5a-desArg and the shorter C5aR-antagonist hC5a-A8 have been shown to crystallize as three-helix bundles^23^,^31^. In agreement with this idea, superimposition of the different three-helix bundle conformations observed for hC5a-desArg^31^ onto the NOX-D20:mC5a complex reveals that helix H1 from these conformations would extensively clash with the G-quadruplex domain (Supplementary Fig. 6e). As a control of the specific binding of NOX-D20 to the ligands hC5a and hC5 in solution, a competitive SPR binding assay showed no binding to hC3a (Supplementary Fig. 6f). Finally, as the C-terminus of C5a extends away from the Spiegelmer molecule and does not participate in the interaction, glycosylations borne by native hC5a on Asn741 (equivalent to Glu745 in mC5a) most probably do not interfere with hC5a binding to NOX-D20.

The capacity of NOX-D20 to inhibit chemotaxis of C5aR1-expressing cells and to antagonize C5a-induced activation of primary human polymorphonuclear leukocytes suggests that the Spiegelmer directly competes with C5aR binding to the anaphylatoxin^22^. Extensive mutational studies led to propose a two-sites binding mode for the C5a:C5aR interaction^32^, according to which positively charged residues located in helices H1–H3 interact with acidic residues and sulfo-tyrosines contained in the C5aR N-terminus, whereas the C5a C-terminus inserts into the C5aR transmembrane domain^33^,^34^,^35^,^36^,^37^. Comparison of the footprint of NOX-D20 onto mC5a with the position of the C5a residues presumably involved in C5aR binding reveals that NOX-D20 strongly overlaps with the first C5aR-binding site (Fig. 5b,c). Although the C5a C-terminus is not masked by the Spiegelmer, blocking C5a access to its primary docking site on C5aR N-terminus appears sufficient to prevent the C5a:C5aR interaction, therefore providing a structural basis for NOX-D20 inhibitory properties.

### Affinity modulation through backbone modifications

**Figure 6:**
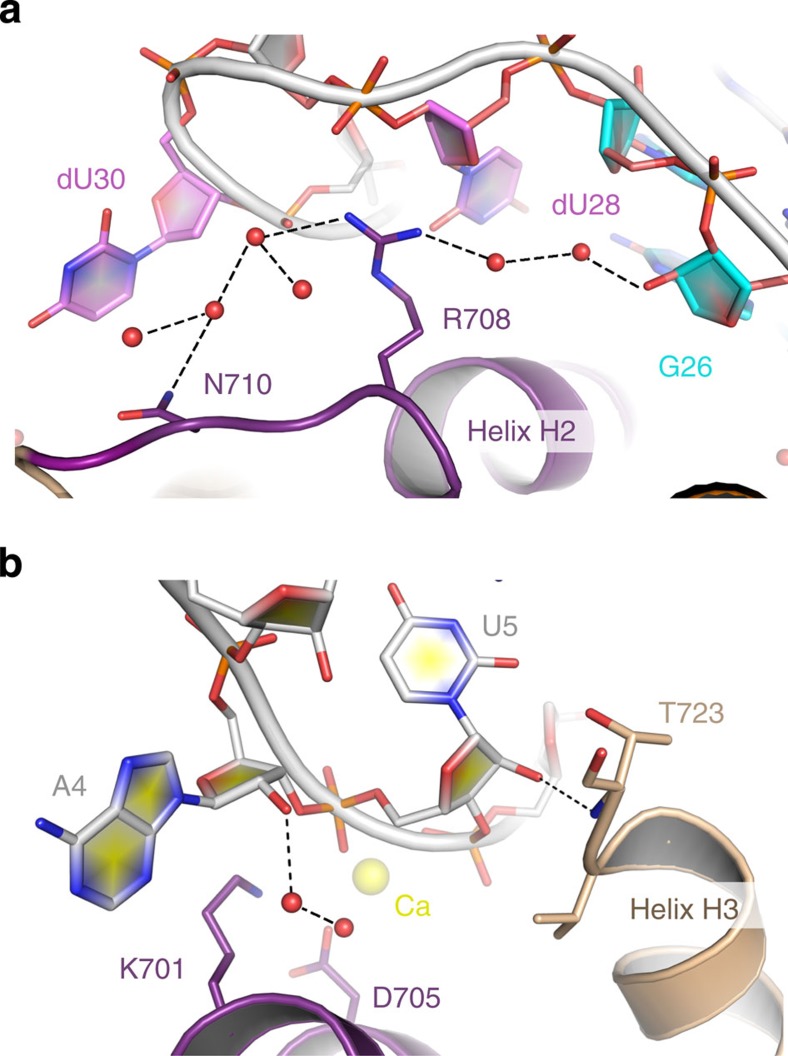
A rationale for NOX-D20 affinity improvement through backbone modifications. (**a**) Introduction of DNA nucleotides at positions 28 and 30 stabilizes the stacking of mC5a Arg708 against the dU28 ribose ring, whereas removal of the 2′-OH group in G26 would destabilize the water network around this interface. (**b**) Modification of A4 and U5 nucleotides directly interferes with mC5a recognition by breaking the U5:Thr723 interaction and by disturbing the water network around Lys701 and Asp705.

The more affine NOX-D20 Spiegelmer was generated from NOX-D19, which only contains L-RNA nucleotides, by introducing L-DNA nucleotides at specific positions^22^. This affinity increase can arise from a fine tuning of the target recognition mode and/or a stronger stabilization of the Spiegelmer active conformation. Detailed analysis of the influence of sugar modifications on NOX-D20 structure reveals that both effects play a role for the modulation of NOX-D20 affinity towards C5a (Table 2). Structure-stabilizing modifications are restricted to the helical stem domain, in regions of strong constriction where the presence of a 2′-OH group on the ribose would loosen the backbone conformation. For example, the absence of 2′-OH groups on the riboses of dU28 and dU30 allows tight packing with the anti-sense strand in the minor groove of the double-helix, thereby shaping the binding interface around the mC5a H2-H3 loop and thus improving target interaction (Fig. 6a). In addition, ribose to deoxyribose modifications leading to decreased target affinity can easily be explained by the NOX-D20:mC5a structure. Such changes can either disrupt direct or water-mediated NOX-D20:mC5a contacts, as observed for A4, U5 or G26 modifications (Fig. 6a,b), or destabilize the base pair stacking and/or the structural domains of the Spiegelmer (G11, G22, C31 and G32 modifications). Thus, the NOX-D20:mC5a structure allows to rationalize the increased affinity of NOX-D20 for mC5a as compared with its all L-RNA predecessor NOX-D19.

## Discussion

The NOX-D20:mC5a and NOX-D20:mC5a-desArg structures reveal for the first time the active conformation adopted by an L-nucleic acid aptamer, in particular of a mixed L-RNA/L-DNA aptamer, in complex with its physiological target. To date, only structures of a short, non-functional, double-stranded L-RNA and of a L/D-RNA racemate were available^38^,^39^. Interestingly, NOX-D20 adopts a complex 3D architecture built on the one hand by a helical stem expanding over 30 Å, and on the other hand, by an intramolecular G-quadruplex stabilized by a central Ca^2+^ ion. This allows the L-aptamer to wrap around its target with tight shape complementarity, thereby providing an affinity for C5a in the picomolar range. The binding mode observed in the structures and confirmed by mutational analysis shows that the Spiegelmer recognizes a large epitope also required for C5aR1 binding and thus provides a rationale for NOX-D20 inhibitory properties. Furthermore, NOX-D20 specificity for human and murine C5a over macaque and rat C5a can be explained by the sole presence of a serine residue interacting directly with the G-quadruplex domain, and a single Cys to Arg mutation is responsible for the increased affinity of NOX-D20 for mC5a as compared with its human counterpart. Finally, our data provide a structural basis for the improved affinity of NOX-D20 that was generated from NOX-D19 by the introduction of six ribo-to-deoxyribonucleotide exchanges^22^. Some of these modifications enhanced target recognition directly, whereas the majority led to intramolecular stabilization, also allowing for a four nucleotides truncation in the terminal helix.

G-quadruplexes are often encountered in nucleic acid aptamers as they provide a much more versatile scaffolding element than simple DNA/RNA duplexes for target recognition, because of the huge diversity of structural motifs that can be obtained by varying the length and connectivity of the loops that bridge the G-tetrads^40^. Well-known structures containing G-quadruplexes are thrombin-binding DNA aptamers^41^. G-quadruplexes are generally stabilized by monovalent cations, mostly Na^+^ and K^+^, which stack either in between two G-tetrad layers (K^+^) or in the plane of the G-tetrads (Na^+^)^27^ (Supplementary Fig. 7). Divalent cations seem to have a more complex role with respect to G-quadruplexes, as they have been reported to destabilize G-quadruplex formation or induce topological transitions in the connectivity between the various G-strands, leading for example to a switch from antiparallel to parallel topology^27^,^42^,^43^. Despite these observations, still only limited structural information is available to study the influence of divalent cations on G-quadruplex structures and to our knowledge, only one structure of a calcium-containing G-quadruplex has been reported so far, the structure of the bimolecular telomeric G-quadruplex d(TG_4_T) from *Oxytricha nova* in a mixed Ca^2+^/Na^+^ environment^43^. The NOX-D20 structure therefore provides the first example of a Ca^2+^-stabilized unimolecular G-quadruplex within an aptamer molecule. The crystal structure also clearly shows that the two G-tetrad layers are twisted by 45°. This different spatial arrangement of the planes may therefore be more compatible with a central Ca^2+^ ion than with a monovalent ion.

Our data suggest that the high affinity of NOX-D20 for C5a results from a bimodular binding mode to which both the G-quadruplex and the stem region contribute. In particular, the helical stem is kept in place by providing one of the guanine nucleotides of the G-quartet, thereby forming a double-pseudoknot-like structure that stabilizes the overall V-shape of the Spiegelmer. Similarly but with less inter-domain interactions, second-generation thrombin-binding aptamers incorporate an additional stem domain to allow for increased affinity and specificity towards their target^44^. Another example of a bimodular aptamer is the recently described IL-6 SOMAmer, which contains DNA nucleotides with modified bases mimicking aromatic side chains^45^. Other RNA aptamers forming duplex-quadruplex junctions and for which the presence of the G-quadruplex is essential for target recognition have also been reported. These include the *in vitro* selected, guanine-rich *sc1* RNA aptamer, which recognizes the RGG peptide of human fragile X mental retardation protein^46^. The RGG peptide binds the aptamer in a pocket at the interface between the stem domain and the G-quadruplex, through tight shape complementarity^46^, similarly to what we observe in our NOX-D20:C5a complex. Another good example is the Spinach aptamer, which binds to and thereby activates the fluorescence of 3,5-difluoro-4-hydroxybenzylidene imidazolinone, a mimic of the intrinsic fluorophore of green fluorescent protein^47^,^48^. In this case, the 3,5-difluoro-4-hydroxybenzylidene imidazolinone ligand stacks in a plan parallel to the first G-tetrad layer, right at the duplex–quadruplex junction^47^,^48^. The present structures reveal that the Spiegelmer NOX-D20 adopts an as complex architectural fold as standard aptamers, thereby achieving a highly efficient target recognition thanks to a strong interconnectivity between two geometrically distinct structural modules.

In summary, we describe the first target-engaged mixed L-RNA/L-DNA Spiegelmer structure which features, besides an expected left-turning helix, an unusual G-quadruplex with a central Ca^2+^ ion, and gives a rationale for affinity improvement by positional ribo-to-deoxyribonucleotide exchanges. From the structure, it becomes clear that binding to a discontinuous epitope on C5a leads to the inhibition of the anaphylatoxin-induced receptor signalling thus supporting the published results from cell-based assays and preclinical research. Further work is warranted to show the exact interaction of the Spiegelmer with C5.

## Methods

### L-Aptamers

The L-aptamers (Spiegelmers) used in this study were manufactured at NOXXON Pharma AG by solid phase synthesis on controlled pore glass support using *tert*-butyl-dimethylsilyl-protected phosphoramidites of L-nucleosides. In previous publications, NOX-D19 and NOX-D20 referred to the oligonucleotides with a 40-kDa Y-shaped methoxy-PEG attached to their 5′-ends via an aminohexyl linker^22^,^49^. In this study, unPEGylated variants were used since PEG may sterically inhibit the formation of crystals. Consequently, affinity measurements and cell-based assays presented here were also done with unPEGylated Spiegelmers. The sequence for NOX-D20 oligonucleotide is L-RNA/L-DNA (40 nt): 5′- GCGAUG(dU)GGUGGU(dG)(dA)AGGGUUGUUGGG(dU)G(dU)CGACGCA(dC)GC-3′ . The sequence of the NOX-D19 oligonucleotide is all L-RNA (44 nt): 5′- GCCUGAUGUGGUGGUGAAGGGUUGUUGGGUGUCGACGCACAGGC-3′ .

### Protein expression and purification

Recombinant hC5a (mutant Cys704Arg), mC5a and mC5a-desArg were expressed recombinantly in bacteria^23^. All proteins were expressed as fusions with an N-terminal thioredoxin tag (Trx-A) followed by a hexahistidine tag and a TEV protease cleavage site (Trx-His_6_-TEV-C5a), in Shuffle T7 Express *E. coli* cells (New England Biolabs). The proteins were purified using a two-step Ni-column affinity chromatography, including removal of the affinity-tag by overnight incubation with TEV protease, followed by a cation exchange chromatography on a Source 15S column (GE Healthcare Life Sciences). The final protein buffer was adjusted to 20 mM HEPES, pH 7.5, 150 mM NaCl before flash freezing in liquid nitrogen and storage at −80 °C until use. Mutants of hC5a and mC5a were generated using the Quick-Change Lightning Site Directed Mutagenesis Kit from Agilent Technologies and all mutants were expressed and purified using the same protocol as for native proteins. Human C3a was expressed recombinantly following the same protocol as for the C5a proteins^50^.

### Crystallization and data collection

Before crystallization, the mC5a and mC5a-desArg samples were concentrated to 20–25 mg ml^−1^ and NOX-D20 was dissolved in water at a final concentration of 2 mM. The mC5a proteins and the L-aptamer were mixed in a 1:1 molar ratio at a final concentration of 1 mM for each component in a final buffer adjusted to 10 mM HEPES, pH 7.5, 137 mM NaCl, 3 mM KCl, 3 mM MgCl_2_, 3 mM CaCl_2_. The mix was allowed to incubate for 2 h at room temperature before setting up crystallization plates. Initial crystallization experiments were carried out in 96-well sitting-drop plates using a MOSQUITO robot (TTP LabTech) and commercial screens from Hampton Research and Molecular Dimensions Ltd. Crystals for both complexes appeared after a few days at 19 °C over a reservoir containing 0.2 M NaCl, 0.1 M Na cacodylate, pH 6.0, 8% (w/v) PEG 8000, for the NOX-D20:mC5a complex, and a reservoir with 0.2 M ammonium acetate, 0.1 M Na acetate pH 4.0, 15% (w/v) PEG 4000, for the NOX-D20:mC5a-desArg complex. In both cases, crystals grew to maximal size within a few weeks. For data collection, the crystals were cryoprotected by soaking into the reservoir solution supplemented with 25–30% glycerol followed by flash freezing in liquid nitrogen. To determine the ion composition in the complex structure, mC5a and NOX-D20 were also mixed as described above in a buffer where KCl had been replaced by 3 mM RbCl and in a buffer where MgCl_2_ had been replaced by 3 mM MnCl_2_. Crystals similar to those observed in the standard buffer grew in the presence of RbCl, whereas no crystals could be obtained when MnCl_2_ was present. For phase determination, 0.3–0.5 μl of osmium hexamine (dissolved in water at 20 mM) were added in the drop containing the crystals of the NOX-D20:mC5a complex (final concentration in the drop between 1.5 and 2.5 mM). Crystals were allowed to incubate with osmium hexamine for 6 h over the reservoir solution before cryoprotection and flash freezing in liquid nitrogen as described above. Native data sets for both complexes, as well as the data sets for the NOX-D20:mC5a crystals derivatized with osmium hexamine or grown with RbCl were collected at 100 K on beamline 911-3 at MAX-lab (Lund, Sweden). The data sets at higher wavelength to identify the ion composition of the asymmetric unit were collected on beamline P13 at PETRA III. All data sets were processed with XDS (X-ray Detection Software)^51^.

### Structure determination

The crystals of the NOX-D20:mC5a complex displayed a P2_1_2_1_2 symmetry and diffracted to a maximal resolution of 1.8 Å (Table 1). Initial attempts to solve the structure were made by performing MR in PHASER^52^ using the structure of mC5a in a four-helix bundle conformation (PDB 4P3A^23^). As hC5a is also able to form a three-helix bundle in its shorter versions hC5a-desArg^31^ and hC5a-A8 (ref. 23), models of mC5a in the different three-helix bundle conformations observed for hC5a-derived proteins were also constructed and used as MR search models (Supplementary Fig. 8). None of these models gave a solution, most probably due to the fact that one molecule of mC5a only represents a small fraction of the asymmetric unit content. Three-dimensional models for the L-aptamer were also constructed in a two-step procedure based on secondary structure predictions (Supplementary Fig. 9). First, a standard D-RNA/D-DNA aptamer was constructed using RNAComposer^53^. In a second step, the mirror-image of this model was obtained by inverting the sign of the Z-coordinate for all atoms of the model. All these models failed to give a MR solution. Despite the presence of 78 phosphate groups arising from the two Spiegelmer molecules expected in the asymmetric unit, experimental phasing using P-SAD from data sets collected at higher wavelength (Table 1) was not successful in providing initial phases either. The NOX-D20:mC5a crystals were therefore derivatized by soaking with various compounds containing heavy atoms, including osmium hexamine, Os(NH_3_)_6_, which mimics almost perfectly a hexahydrated Mg^2+^ and has therefore been extensively used to solve large RNA 3D structures^54^. Anomalous data sets extending to 1.8 Å resolution were collected for the Os(NH_3_)_6_-derivatized crystals and the structure was solved by SAD phasing in PHENIX.AUTOSOLVE^55^ (Table 1, Supplementary Figs 1 and 2). Sixteen refined Os(NH_3_)_6_ sites were identified. The overall figures of merit after SAD phasing and after density modification using RESOLVE were 0.48 and 0.78, respectively (35–2.5 Å resolution cutoff).

The quality of the initial electron density maps allowed to unambiguously identify the elements composing the asymmetric unit, that is, three molecules of mC5a and two molecules of NOX-D20 (Supplementary Fig. 3). In particular, it was readily visible that mC5a adopts a four-helix bundle conformation and the three mC5a molecules were placed manually in the electron density map using the structural model available for mC5a^23^. The density patterns left accounted for two molecules of Spiegelmer arranging in an upside-down manner without apparent discontinuity in the base-pair stacking between their helical stems. Careful inspection of the density allowed for positioning of the 5′ and 3′ ends of both Spiegelmer molecules. As the Spiegelmer is built from non-conventional L-nucleotides, geometry files for each of the eight L-RNA/L-DNA nucleotides were parameterized manually to allow rebuilding and refinement of the Spiegelmer molecules using standard programmes. The 40 nucleotides composing each NOX-D20 molecule were then placed manually one by one in the density and refinement of the model was carried out using conventional procedures, after having defined the geometrical parameters for the phosphate bonds linking each pair of nucleotides in PHENIX.REFINE^55^. After an initial round of refinement, the obtained model was used to perform MR in the native data set, which displayed slightly better statistics (Table 1). The model was then further improved by manual rebuilding in COOT^56^ and energy minimization in PHENIX.REFINE^55^ using individual isotropic Atomic Displacement Parameters (ADP) and Translation–Libration–Screw (TLS) parameterization. Non-Crystallographic Symmetry restraints were not imposed as they were observed to increase the R-factor values during refinement. The final model yielded *R*_work_ and *R*_free_ values of 16.70% and 19.26%, respectively, and was used as a MR search model to solve the structure of the NOX-D20:mC5a-desArg complex. Refinement of the NOX-D20:mC5a-desArg model was carried out by alternating between cycles of manual rebuilding in COOT^56^ and cycles of energy minimization with PHENIX.REFINE^55^ using individual isotropic ADP, TLS refinement, and the same geometry restraints as for the NOX-D20:mC5a complex, again without imposing any Non-Crystallographic Symmetry restraints. The final model yielded *R*_work_ and *R*_free_ values of 16.26% and 19.94%, respectively. The quality of both models was assessed with MOLPROBITY^57^. The Ramachandran statistics for the NOX-D20:mC5a and NOX-D20:mC5a-desArg complexes are, respectively: outliers, 0.5%/0%; allowed, 2.3%/2.5%; favoured 97.2%/97.5%. Simulated annealing refinement was performed in PHENIX.REFINE^55^ after removal of the Ca^2+^ ions in the final model using a start temperature of 1,500 K and cooling down to 300 K. Refinement with tight metal-O geometry restraints was performed by adding back either Ca^2+^ or K^+^ ions in the model obtained after simulated annealing refinement and by doing energy minimization in PHENIX.REFINE^55^ using individual isotropic ADP, TLS refinement and imposing the Ca–O or K–O distances to 2.43 Å±0.05 *σ* or 2.81 Å±0.05 *σ*, respectively, during the refinement step. All figures were produced with PyMOL v.1.7 (http://www.pymol.org). C5a sequence alignment was performed in MULTIALIN^58^ and the sequence conservation analysis was done in ALINE^59^.

### Biacore direct binding assay format

The Biacore 2000 instrument was set to a constant temperature of 37 °C. The system was cleaned using the DESORB method before the start of each experiment/immobilization of a new chip. After docking a maintenance chip, the instrument was consecutively primed with desorb solution 1 (0.5% SDS), desorb solution 2 (50 mM glycine, pH 9.5) and HBS-EP pH 7.4 buffer. Finally, the system was primed with HBS-EP pH 7.4 buffer (GE Healthcare). Human and murine C5a and their mutants were immobilized by amine coupling procedure on the CM5 sensor chips (flow cells 2–4, whereas flow cell 1 served as dextran surface control). A volume of 100 μl of a 1:1 mixture of 0.4 M EDC (1-ethyl-3-(3-dimethylaminopropyl) carbodiimide in H_2_O; GE, BR-1000-50) and 0.1 M NHS (*N*-hydroxysuccinimide in H_2_O; GE, BR-1000-50) were injected using the QUICKINJECT command at a flow of 10 μl min^−1^. The C5a proteins were diluted to a concentration of 1 μg ml^−1^ in 10 mM sodium acetate, pH 5.5, with 1 μM NOX-D20 and subsequently C5a was immobilized covalently up to approximately 500 response units (RUs) at a flow of 10 μl min^−1^. The flow cells were blocked by an injection 70 μl of 1 M ethanolamine hydrochloride (GE, BR-1000-50) at a flow of 10 μl min^−1^.

On the day of the sample measurement, the sensor chip was primed twice with degassed physiological running buffer (20 mM Tris pH 7.4; 150 mM NaCl; 5 mM KCl, 1 mM MgCl_2_ and 1 mM CaCl_2_) and equilibrated at a flow of 50 μl min^−1^ until the baseline appeared stable. Before sample measurement, the chip underwent at least three injection and regeneration cycles whereby regeneration was performed by injecting 30 μl of 5 M NaCl at a flow of 30 μl min^−1^. Stabilization time of baseline after each regeneration cycle was set to 1 min at 30 μl min^−1^. A concentration series (500; 250; 125; 62.5; 31.3; 15.6; 7.8; 3.9; 1.95; 0.98; 0.49; 0.24; 0.12; 0.06; 0 nM) of NOX-D19 or NOX-D20 was prepared in physiological running buffer and injected starting with the lowest concentration. Regeneration was performed after each measurement. In all experiments, the analysis was performed at 37 °C using the KINJECT command defining an association time of 240 s and a dissociation time of 240 s at a flow of 30 μl min^−1^. The assay was double referenced, whereas flow cell 1 served as (blocked) surface control (bulk contribution) and a series of buffer injections without analyte determined the bulk contribution of the buffer itself on the loaded flow cells. At least one Spiegelmer concentration was injected a second time at the end of the experiment to monitor the regeneration efficiency and chip integrity during the experiments. Data analysis and calculation of dissociation constants (*K*_d_) by fitting the data to a 1:1 Langmuir algorithm were done with the BIAevaluation 3.1.1 software (BIACORE AB) with a constant refractive index and an initial mass transport coefficient *k*_t_ of 1 × 10^7^ (RU M^−1^ s^−1^).

### Biacore competitive binding assay format

For competitive analysis, mC5a or hC5a(Cys704Arg), respectively, was immobilized on a CM5 sensor chip as described above. Co-injection of NOX-D20 with single C5a proteins will result in a competition between the chemokine in solution and the immobilized C5a on the sensor chip for NOX-D20 binding, thus leading to a reduction in signal. The reduction of the signal can be evaluated at a predefined report point, that is, at the end of the dissociation time (see Supplementary Fig. 5a). For detailed analysis, a concentration series of the respective C5a, C5 or C3a protein was injected. Potential unspecific binding of the individual proteins to the dextran matrix or the immobilized C5a was monitored by a control flow cell without the C5a, as well as by an injection of the respective proteins without NOX-D20.

### Influence of cations on NOX-D20 binding

Murine C5a was immobilized as described above. First 100 nM of NOX-D20 was co-injected with a concentration series (16; 8; 4; 2; 1; 0.5; 0.25; 0.125; 0.0625; 0.0313; 0 (2 × ) mM) of EDTA in physiological running buffer (20 mM Tris, pH 7.4; 150 mM NaCl; 5 mM KCl, 1 mM MgCl_2_ and 1 mM CaCl_2_). The Mg^2+^- and Ca^2+^-dependent binding of NOX-D20 to immobilized mC5a was addressed by co-injection of 100 nM NOX-D20 with a concentration series (16; 8; 4; 2; 1; 0.5; 0.25; 0.125; 0.0625; 0.0313; 0 (2 × ) mM) of MgCl_2_ or CaCl_2_, respectively, in physiological running buffer without the corresponding divalent ion. To confirm the influence of Ca^2+^ ions also on the stability of the complex, CaCl_2_ was only titrated in the running/dissociation buffer, while kept at a physiological concentration (1 mM) in the injection/association buffer. To determine the presence of potassium ions, KCl was also titrated down in affinity measurements starting at the physiological concentration of 5 mM (5; 2.5; 1.25; 0.625; 0.313; 0 mM). This was followed by the titration of LiCl (40; 20; 10; 5; 2.5; 1.25; 0.625; 0.313; 0.156; 0 mM) in the absence of KCl to replace potentially present trace amounts of K^+^ and break the G-quartet if potassium was required for proper folding. Data analysis and calculation of association rate constant (*k*_a_) was performed as described above. All final data were analysed using Prism 5 software (GraphPad Software).

### Chemotaxis assay

Chemotaxis assays were performed in mouse BA/F3 cells stably transfected using a plasmid coding for the human C5a receptor, hC5aR1 (ref. 22). The hC5aR1-expressing BA/F3 cells were stimulated with recombinant mutated mC5a and hC5a proteins at varying concentrations as indicated. Recombinant WT mC5a or recombinant hC5a(Cys704Arg) were used as controls, respectively. Commercially available recombinant WT mC5a and hC5a (R&D Systems) were also tested for comparison. Chemotaxis was followed in Transwell plates with 5 μm pores at 37 °C for 3 h in HBH buffer. For quantification of migrated cells, 50 μM resazurin in PBS was added and incubated at 37 °C for 2.5 h. Fluorescence was measured at 590 nm (excitation wavelength 544 nm). Data were analysed using Prism 5 software (GraphPad Software).

## Author contributions

L.Y. prepared all recombinant proteins, performed crystallization, data collection and structure determination/refinement. C.M. performed the SPR measurements. K.H. provided potential two-dimensional structures as starting points for the structural analysis. L.Y., C.M. and A.V. designed the experiments and analysed the data. L.Y., A.V., K.H., C.M., S.K. and G.R.A. wrote the paper.

## Additional information

**Accession codes:** Coordinates and structure factors for the NOX-D20:mC5a and NOX-D20:mC5a-desArg complexes have been deposited in the Protein Data Bank with accession codes 4WB2 and 4WB3, respectively.

**How to cite this article:** Yatime, L. *et al*. Structural basis for the targeting of complement anaphylatoxin C5a using a mixed L-RNA/L-DNA aptamer. *Nat. Commun.* 6:6481 doi: 10.1038/ncomms7481 (2015).

## Supplementary Material

Supplementary Information Supplementary Figures 1-9 and Supplementary References

## Figures and Tables

**Table 1:** Data collection and refinement statistics for the native and anomalous data sets.

	**NOX-D20:mC5a (native)**	**Os(NH**_**3**_**)**_**6**_ **data set**	**RbCl data set**	**High wavelength data set**	**NOX-D20:mC5a-desArg (native)**
*Data collection*					
Space group	P2_1_2_1_2	P2_1_2_1_2	P2_1_2_1_2	P2_1_2_1_2	P2_1_2_1_2
*Cell dimensions*					
*a*, *b*, *c* (Å)	46.47, 282.25, 45.90	45.69, 283.24, 45.92	45.76, 282.15, 45.78	45.78, 282.88, 45.85	45.84, 282.63, 45.76
*α*, *β*, *γ* (°)	90, 90, 90	90, 90, 90	90, 90, 90	90, 90, 90	90, 90, 90
Wavelength (Å)	0.91	1.1403	0.81	2.4984	1.0
Resolution (Å)* †	50–1.8 (1.9–1.8)	50–1.8 (1.9–1.8)	50–2.5 (2.6–2.5)	50–2.5 (2.6–2.5)	50–2.0 (2.1–2.0)
*R*_meas_	6.5 (93.3)	9.0 (72.0)	16.8 (85.0)	8.5 (38.1)	9.8 (59.8)
*I*/σ*I*	21.62 (2.98)	17.65 (3.0)	9.23 (1.84)	18.52 (6.10)	12.01 (2.72)
Completeness (%)	99.9 (100)	100 (100)	99.9 (99.9)	86.7 (46.4)	99.8 (100)
Redundancy	9.8 (8.2)	8.3 (8.4)	3.7 (3.7)	6.8 (5.9)	4.7 (4.8)
					
*Refinement*					
Resolution (Å)	35–1.8				35–2.0
No. reflections	56,027				40,265
*R*_work_/*R*_free_	16.70/19.26				16.26/19.94
*No. of atoms*					
Protein	1,755				1,670
RNA/DNA	1,716				1,716
Ligand/ion	32				20
Water	508				373
*B-factors*					
Protein	36				38
RNA/DNA	32				37
Ligand/ion	47				45
Water	39				36
*R.m.s. deviations*					
Bond lengths (Å)	0.006				0.007
Bond angles (°)	1.366				1.469

^*^One crystal for each structure was used for data collection and structure determination.

^†^Values for the highest resolution shell are shown in parentheses.

**Table 2:** Effect of positional L-ribonucleotide to L-deoxyribonucleotide exchanges on C5a recognition.

**NOX-D19**	**1.37**±**0.22 nM (44 nt all-RNA NOX-D20 predecessor)**			
**Modified nucleotide**	**X-fold effect on C5a affinity**	**Increase/decrease by**	**Structural basis for the observed effect upon –OH to –H shift**	
A4	**7.2x Decrease**	Both	Target binding	Destabilization of H_2_O network around the target
U5	**6.1x Decrease**	Off-rate	Target binding	Hydrogen bond to Thr723 main chain broken
U7	**2.0x Increase**	On-rate	Spiegelmer fold	Shift to 4′-exo ribose conformation
G11	**5.1x Decrease**	Both	Spiegelmer fold	-OH interaction with N7 of dG14 disrupted
G14	**1.4x Increase**	On-rate	Spiegelmer fold	Shift to 4′-exo ribose conformation
A15	**1.6x Increase**	On-rate	Spiegelmer fold	Stabilization of the dG14 *syn* conformation
G22	**12.5x Decrease**	On-rate	Spiegelmer fold	Destabilization of G-quadruplex loop region
G26	**1.7x Decrease**	Off-rate	Target binding	Destabilization of H_2_O network around the target
U28	**1.9x Increase**	Both	Both	Stabilization of loop region around R708-N710
U30	**1.6x Increase**	Both	Both	Stabilization of loop region around R708-N710
C31	**12.0x Decrease**	Off-rate	Spiegelmer fold	Destabilization of H_2_O network around Mg^2+^
G32	**10.2x Decrease**	Off-rate	Spiegelmer fold	Destabilization of the Domains 1-2 interface
C38	**1.6x Increase**	Off-rate	Target binding	Stabilization of H_2_O network around the target

Influence of the removal of the ribose 2′-OH group on the Spiegelmer affinity for C5a as evaluated by SPR^22^ and detailed analysis of the influence of backbone modifications on the structural properties of NOX-D20.
