# Supplementary material for: Structural basis for the targeting of complement anaphylatoxin C5a using a mixed L-RNA/L-DNA aptamer
Source: Nat Commun. 2015 Apr 22;6:6481. doi: 10.1038/ncomms7481 (PMC4423239; doi:10.1038/ncomms7481)
Supplement: Supplementary Information — Supplementary Figures 1-9 and Supplementary References [file ncomms7481-s1.pdf]

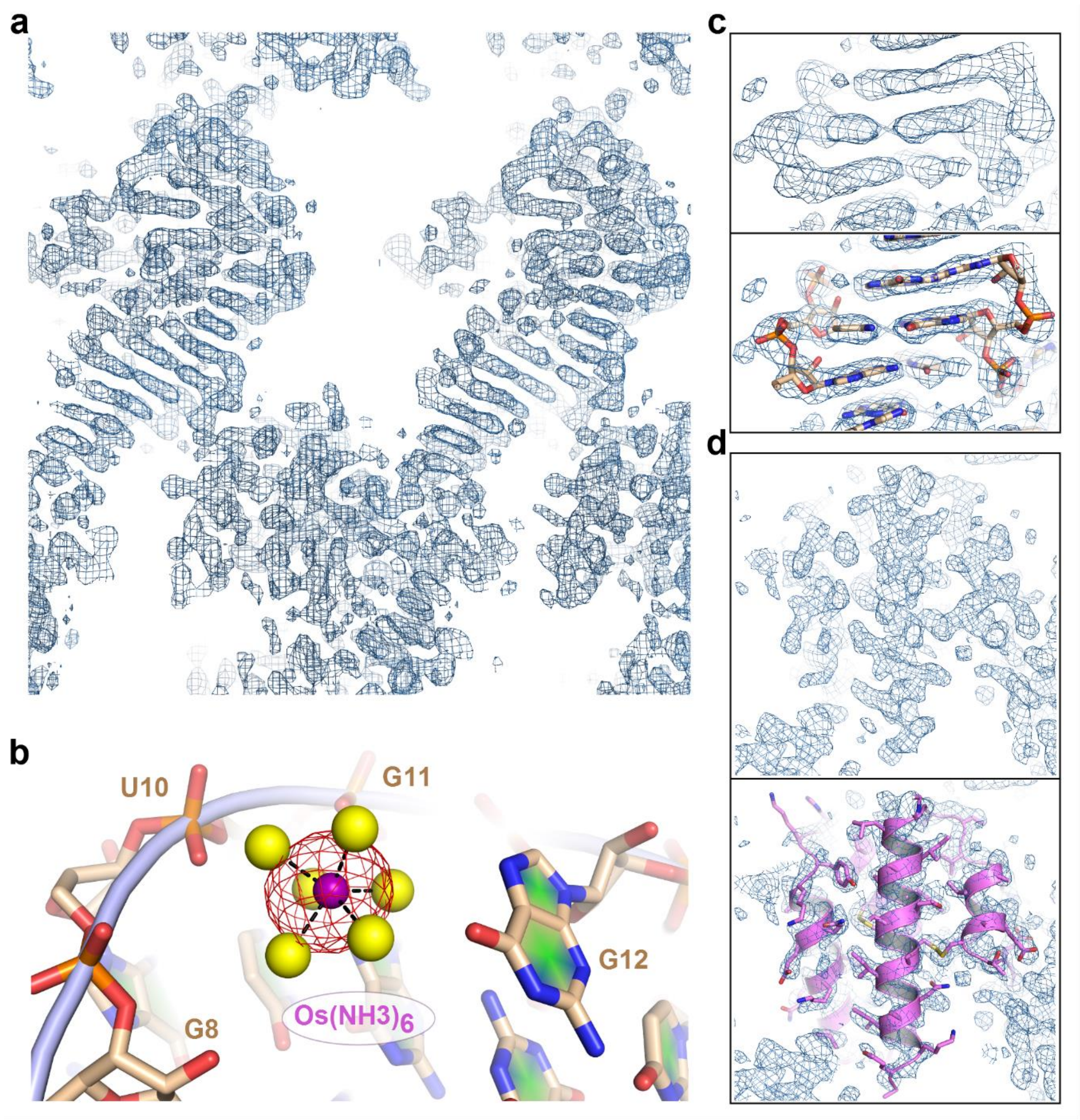

Supplementary Fig. 1. Initial electron density maps for the NOX-D20:mC5a complex obtained after SAD-phasing. **(a)** Initial experimental electron density map obtained after SAD-phasing and density modification in PHENIX.AUTOSOL[1] using the anomalous dataset collected on the $Os(NH_3)_6$-derivatized crystals. The map is shown as blue mesh and contoured at 1σ. **(b)** Anomalous difference electron density map using SAD phases displayed as red mesh and contoured at 6σ, showing the position of one of the $Os(NH_3)_6$ molecules. The final model does not contain any $Os(NH_3)_6$ molecules as it was refined against the native dataset. **(c)** Zoom-in on the initial electron density map displayed in panel **a** around the stem region of the aptamer. The stacks of base pairs are clearly visible. The final model is superimposed in the lower part for comparison. **(d)** Zoom-in on the initial electron density map displayed in panel **a** around one of the mC5a molecules. The four-helix bundle conformation is already clearly visible in the initial map. The final model is superimposed in the lower part for comparison.

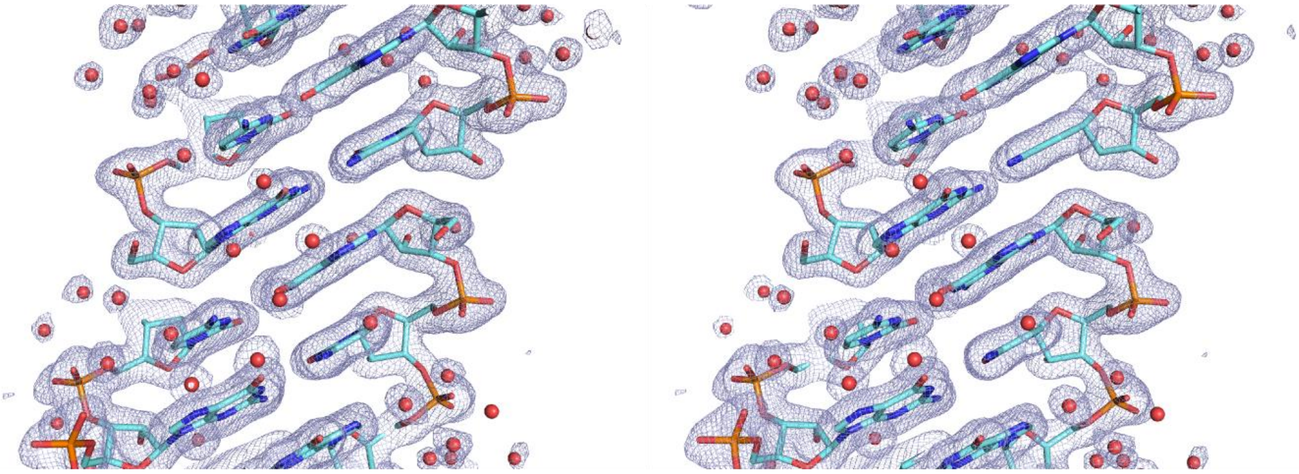

Supplementary Fig. 2. Stereo image of a region of the final $2mF_o$-DF_c electron density map. The map is contoured at 1σ and centered on the stem domain of the Spiegelmer molecule.

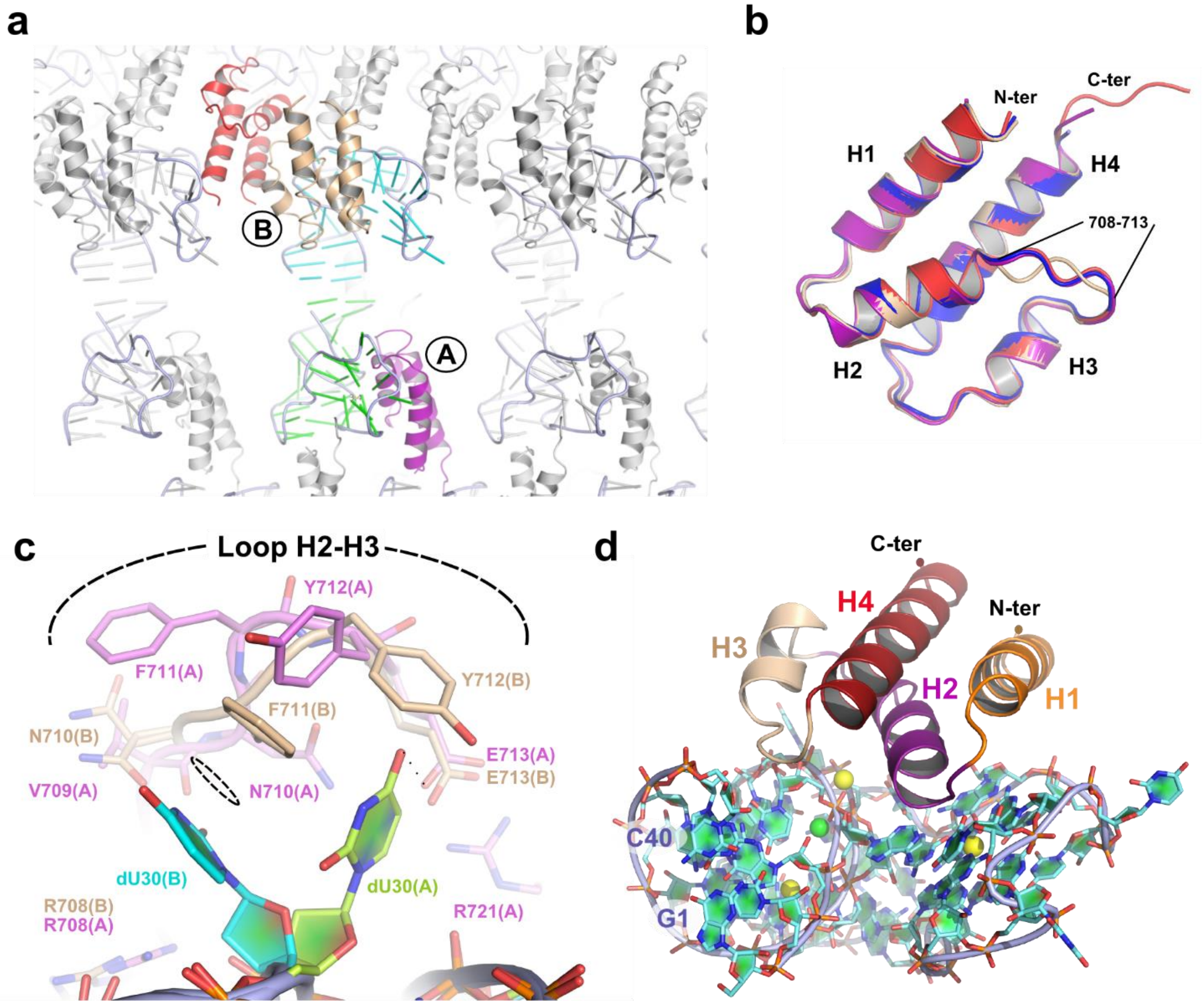

Supplementary Fig. 3. Composition of the asymmetric unit in the NOX-D20:mC5a crystals and structure of the NOX-D20:mC5a-desArg complex. (a) Packing in the NOX-D20:mC5a crystals. The asymmetric unit contains three mC5a molecules but only two NOX-D20 molecules. The two NOX-D20:mC5a complexes shown as A and B (green and purple colors for A, blue and beige colors for B) are equivalent. The red mC5a molecule stacks in between the different layers of complexes and helps maintaining the crystal packing. **(b)** Superimposition of the three mC5a molecules present in the asymmetric unit (purple, beige and red) with the structure of isolated mC5a in dark blue[2]. The region of divergence corresponding to loop H2-H3 is indicated. **(c)** Superimposition of the two equivalent NOX-D20:mC5a complexes present in the asymmetric unit around the region where the two complexes differ. The conformational variation of loop H2-H3 in mC5a is linked to the movement of dU30 in the Spiegelmer, with its base either interacting (long-range) with Glu713 side chain (complex A) or stacking against Phe711 aromatic ring (complex B). **(d)** Structure of the NOX-D20:mC5a-desArg complex at 2.0 Å resolution.

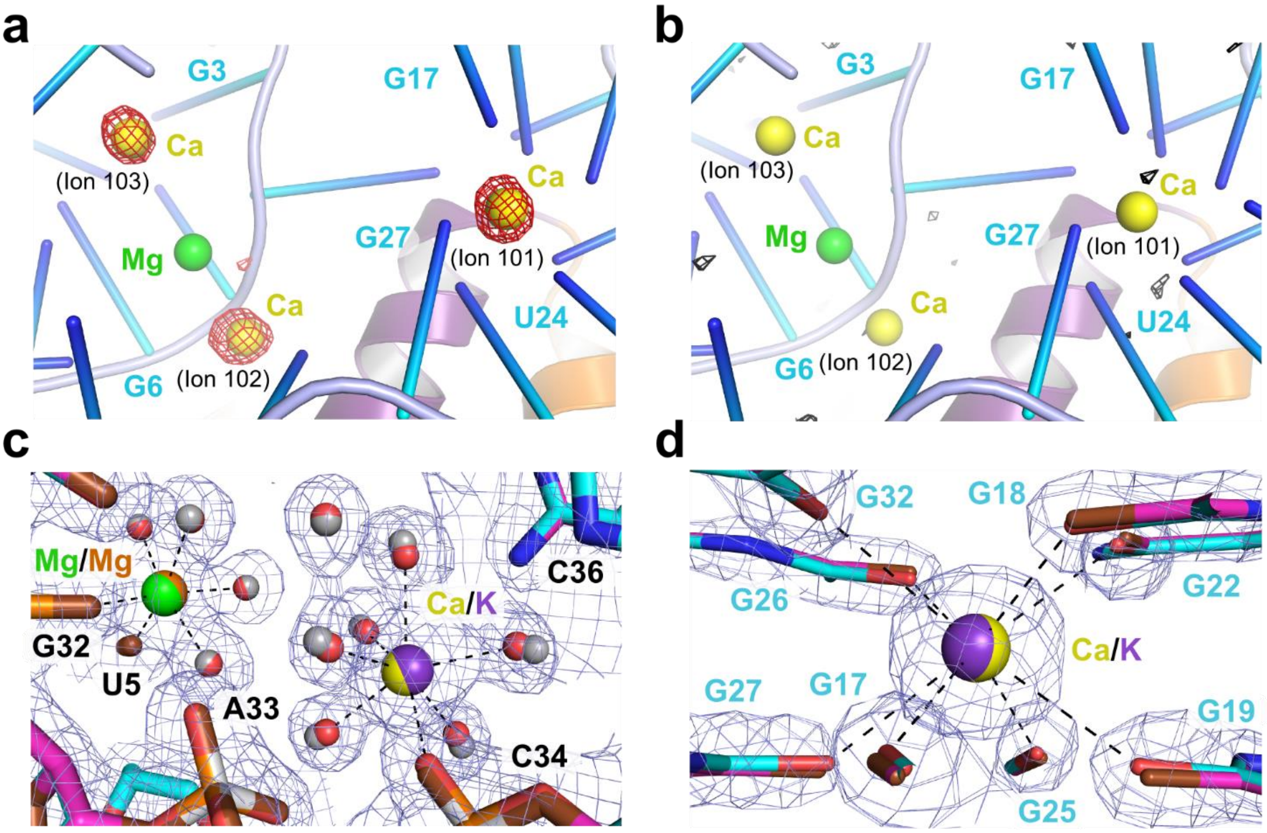

e

Average metal-oxygen distance (Å)	Ion 101 Chain D (G-quartet)	Ion 101 Chain E (G-quartet)	Ion 102 Chain D	Ion 102 Chain E	Ion 103 Chain D	Ion 103 Chain E
in the final model	2.52 ± 0.05	2.49 ± 0.04	2.45 ± 0.11	2.43 ± 0.09	2.47 ± 0.20	2.47 ± 0.11
after Ca^{2+} removal followed by simulated annealing refinement	2.52 ± 0.05	2.50 ± 0.03	2.49 ± 0.07	2.43 ± 0.07	2.49 ± 0.08	2.47 ± 0.11
after adding back Ca^{2+} and imposing tight geometry restraints on the Ca-O distance	2.45 ± 0.03	2.45 ± 0.02	2.43 ± 0.06	2.41 ± 0.05	2.42 ± 0.05	2.41 ± 0.03
after adding back K^+ and imposing tight geometry restraints on the K-O distances	2.62 ± 0.03	2.62 ± 0.03	2.56 ± 0.07	2.60 ± 0.08	2.64 ± 0.08	2.68 ± 0.05
from reported structures in the Cambridge Structural Database	**Ca-O = 2.43 ± 0.11 Å**			**K-O = 2.81 ± 0.10 Å**		

Supplementary Fig. 4. Identification of the ions present in the NOX-D20:mC5a complex. (a) Electron density map (red mesh, contoured at 6σ) calculated as an anomalous difference Fourier map using phases and figure of merit from the final atomic model refined against the native dataset and anomalous differences from the dataset collected on native crystals at a wavelength of 2.498 Å. **(b)** Electron density map (black mesh, contoured at 3σ) calculated as an anomalous difference Fourier map using phases and figure of merit from the final atomic model refined against the native dataset and anomalous differences from the dataset collected from the RbCl-derivatized crystals at a wavelength of 0.81 Å. **(c)** and **(d)** $2mF_o\text{-}DF_c$ maps (blue or gray mesh, contoured at 1σ and 3σ in panel **c** and **d**, respectively) and models obtained after removing the Ca^{2+} ions from the final model, performing a round of refinement in

PHENIX.REFINE[1] using simulated annealing (SA) at 1500 K to remove model bias, and finally reintroducing the ions (Ca^{2+} or K^{+}) and imposing tight geometry restraints on the ion-oxygen distances during refinement (Ca-O = 2.43 Å ± 0.05 σ or K-O = 2.81 Å ± 0.05 σ). In the final model obtained with Ca^{2+}, the RNA nucleotides are colored in cyan, red and orange; Mg^{2+}, Ca^{2+} and water molecules are displayed as green, yellow and red spheres, respectively. In the final model obtained with K^{+}, the RNA nucleotides are colored in pink, brown and white; Mg^{2+}, K^{+} and water molecules are displayed as orange, purple and gray spheres, respectively. **(e)** Comparison of the average metal-oxygen distances (± standard deviation) observed for the 6 putative calcium ions in the NOX-D20:mC5a structure i) in the final, unrestrained model, ii) in the model without Ca^{2+} obtained after SA refinement, iii) and iv) in the models where the ions (Ca^{2+} or K^{+}) have been added back after SA refinement and tight geometry restraints have been imposed on the ion-O distances during the refinement step. The mean values for Ca-O and K-O distances as extrapolated from the Cambridge Structural Database[3] are indicated for comparison.

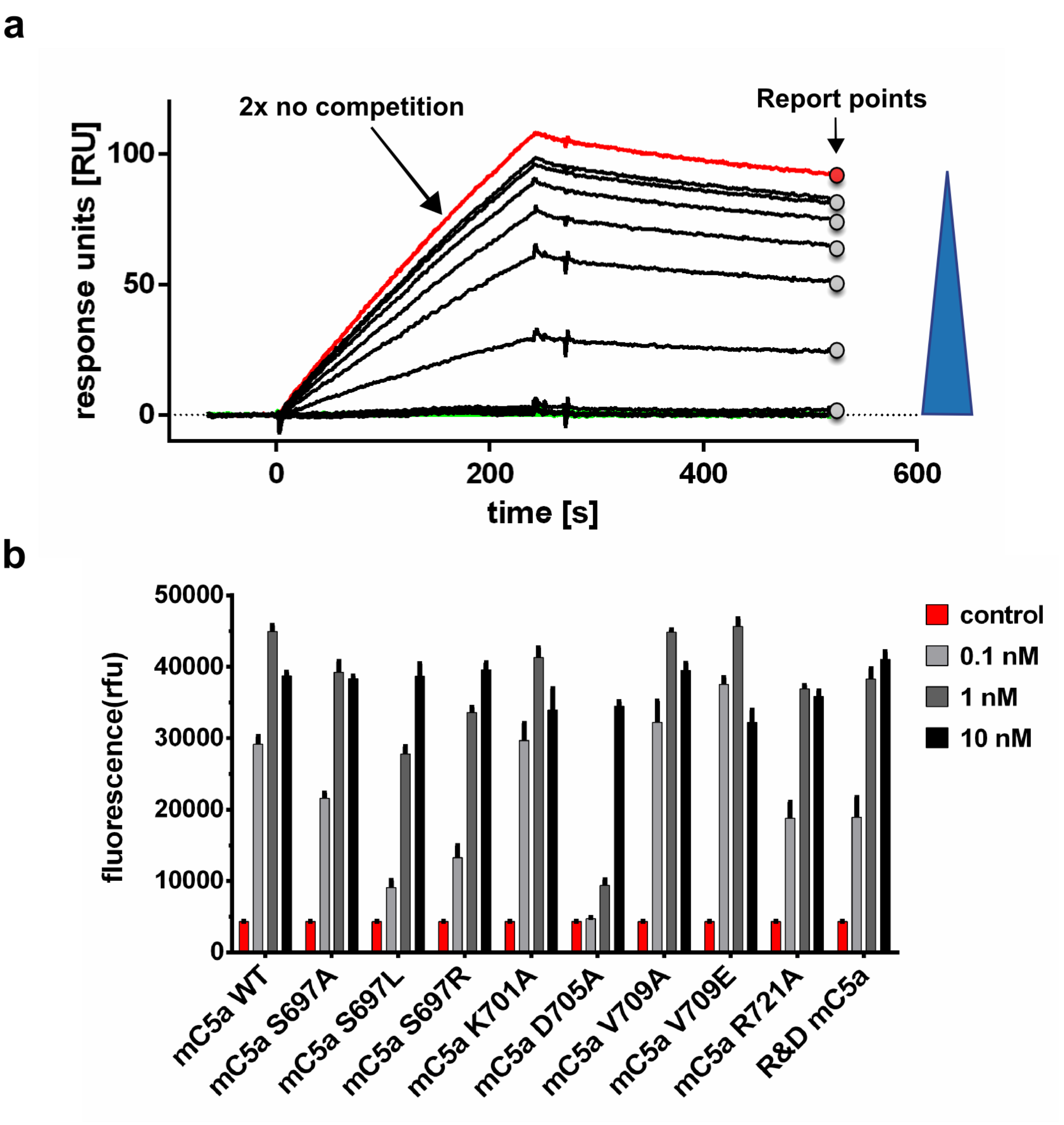

Supplementary Fig. 5. Analysis of mC5a mutants binding to NOX-D20. (a) Example for a competitive SPR measurement with increasing competitor concentrations (top to bottom). **(b)** Analysis of the bioactivity of the murine C5a WT and mutants at different concentrations by a cell-based chemotaxis assay on hC5aR1-expressing BA/F3 cells. Commercially available recombinant mC5a (R&D) was used as a control.

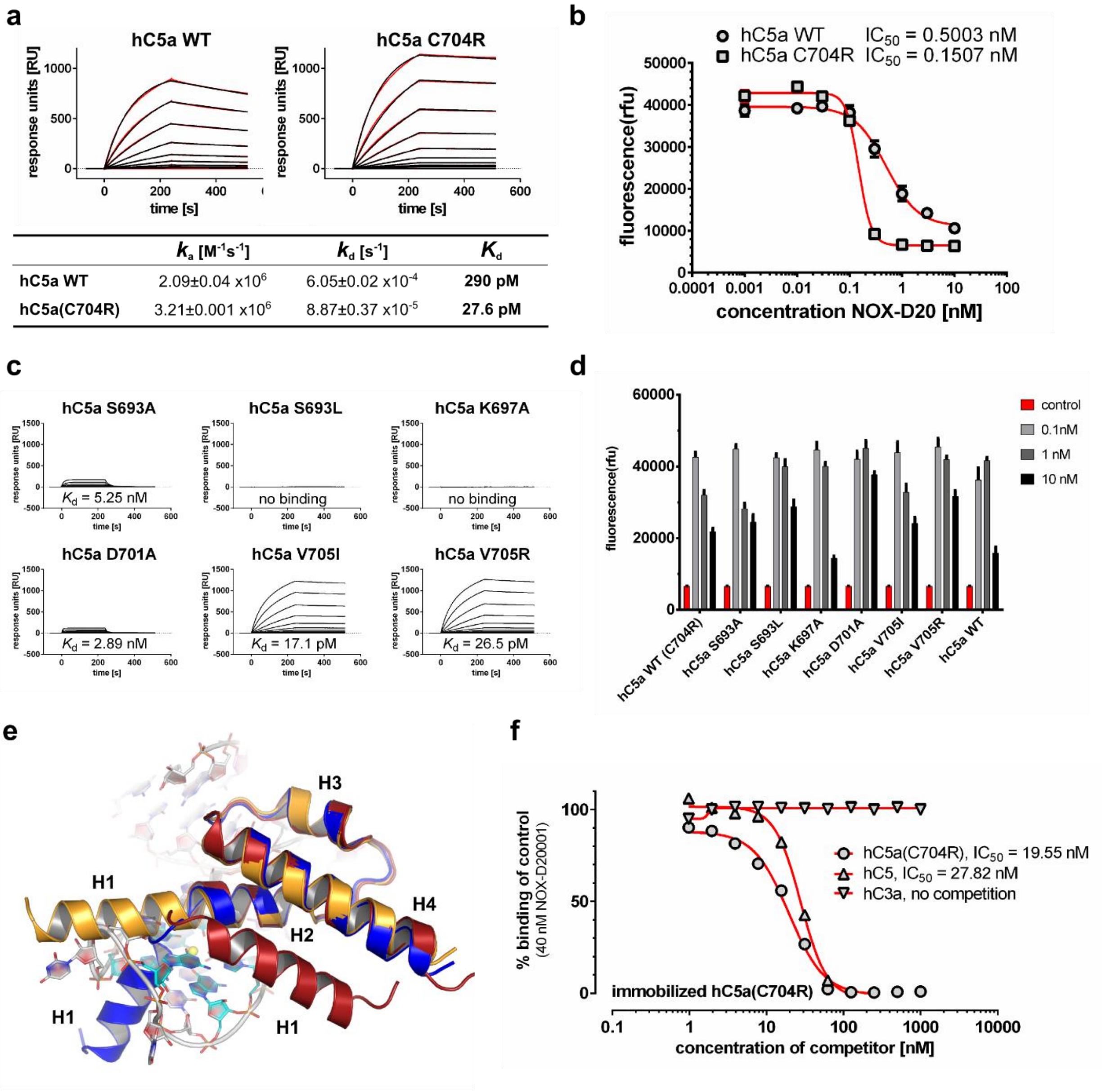

Supplementary Fig. 6. Analysis of hC5a mutants binding to NOX-D20. (a) Comparison of the NOX-D20 binding affinities of our recombinant hC5a (mutant Cys704Arg) with commercially available WT recombinant hC5a. **(b)** Inhibition of hC5a(WT)- and hC5a(Cys704Arg)-induced chemotaxis on hC5aR1-expressing BA/F3 cells by increasing concentrations of NOX-D20. The IC_{50} values for each protein are indicated in the inset. **(c)** Direct measurement of the affinity of NOX-D20 for hC5a(Cys704Arg)-based mutants by SPR. **(d)** Chemotactic activity of WT hC5a and hC5a(Cys704Arg)-based mutants on hC5aR1-expressing BA/F3 cells. **(e)** Superimposition of the two three-helix bundle conformations observed for hC5a-desArg[4] (in blue and orange) onto the NOX-D20:mC5a complex (mC5a in red), revealing that in these conformations hC5a N-terminal helix would clash with the G-quadruplex domain of NOX-D20. **(f)** Competition of NOX-D20 binding to immobilized hC5a(Cys704Arg) by hC5a(Cys704Arg), hC5 and hC3a measured by SPR (analysis by report point at 240 s of dissociation).

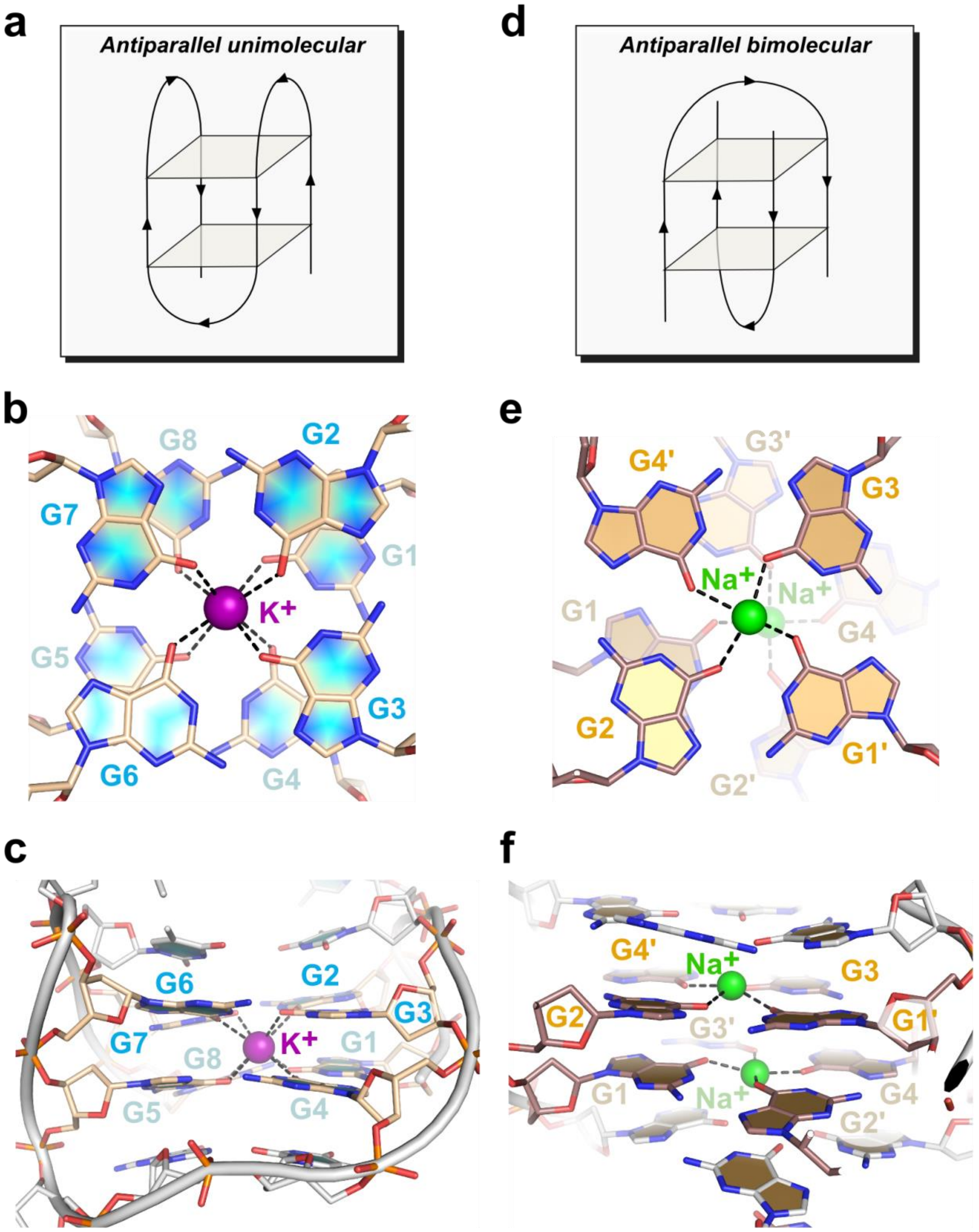

Supplementary Fig. 7. Examples of G-quadruplexes stabilized by monovalent cations. (a) Topological diagram of the G-quadruplex domain of the thrombin-binding aptamer stabilized by potassium[5] (PDB_ID 4DII). **(b)** Top-view of the G-quadruplex of thrombin-binding aptamer showing how the central K^+ ion stabilizes the stacking of the 2 G-tetrads. **(c)** Lateral view of the G-quadruplex of thrombin-binding aptamer. The central K^+ lies on a layer in between the 2 G-tetrads. **(d)** Topological diagram of the bimolecular G-quartet formed by *Sterkiella nova* telomeric DNA stabilized by sodium[6] (PDB_ID 1JB7). **(e)** Top-view of the *S. nova* telomeric G-quartet showing how each G-tetrad is stabilized by a central Na^+ ion. **(f)** Lateral view of the *S. nova* telomeric G-quartet showing the Na^+ ions on the same plane as the G-tetrads.

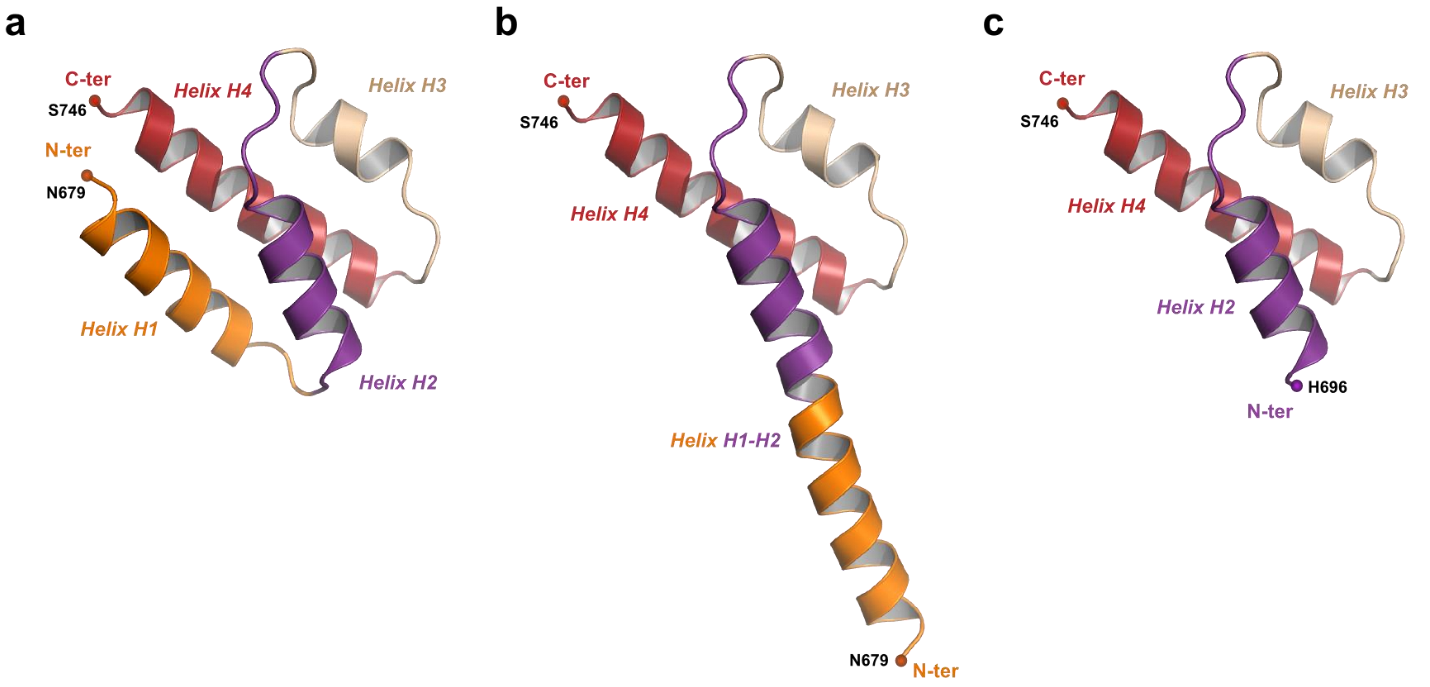

Supplementary Fig. 8. mC5a search models for molecular replacement. (a) Four-helix bundle model for mC5a based on the structure of isolated mC5a[2]. **(b)** Three-helix bundle model with an extended N-terminal helix for mC5a based on the structure of human C5a-desArg[4]. **(c)** Three-helix bundle model corresponding to the conserved helical core between all C5a proteins based on the structure of human C5a-desArg[4].

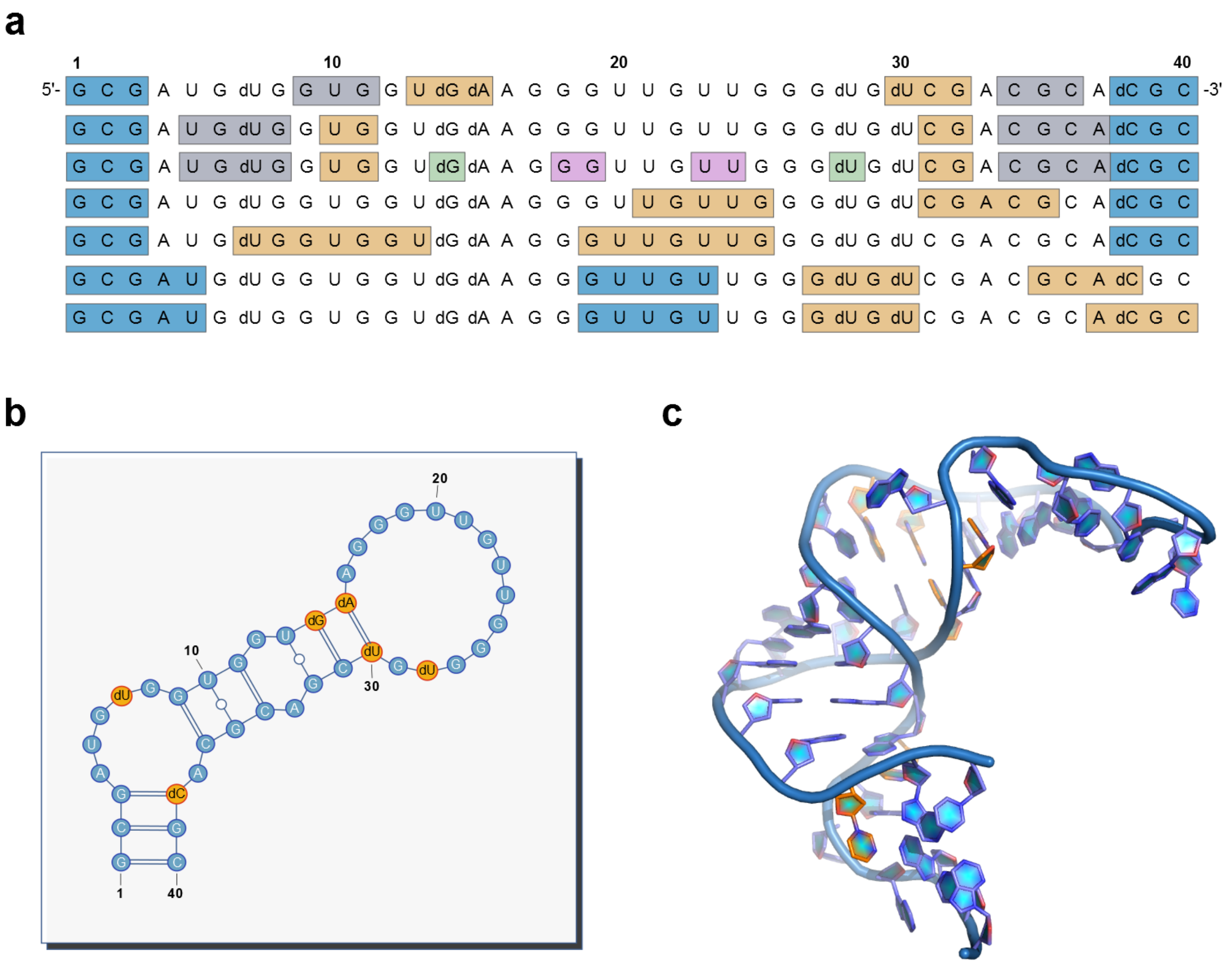

Supplementary Fig. 9. Secondary structure prediction for NOX-D20. (a) Possible secondary structures for the Spiegelmer NOX-D20 predicted from its nucleotide sequence using ViennaRNA[7]. **(b)** Topological representation of the first putative secondary structure model for NOX-D20 (model with the lowest free energy). **(c)** Three-dimensional model corresponding to the secondary structure displayed in panel **b** as generated by taking the mirror-image of a D-configured model built with RNAComposer[8].

Supplementary references

1 Adams, P.D. *et al.* PHENIX: building new software for automated crystallographic structure determination. *Acta Cryst. D* **58**, 1948-1954 (2002).

2 Schatz-Jakobsen, J.A. *et al.* Structural and functional characterization of human and murine C5a anphylatoxins. *Acta Cryst. D* **70** (2014).

3 Zheng, H., Chruszcz, M., Lasota, P., Lebioda, L. & Minor, W. Data mining of metal ion environments present in protein structures. *J. Inorg. Biochem.* **102**, 1765-1776 (2008).

4 Cook, W.J., Galakatos, N., Boyar, W.C., Walter, R.L. & Ealick, S.E. Structure of human desArg-C5a. *Acta Cryst. D* **66**, 190-197 (2010).

5 Krauss, I.R., Pica, A., Merlino, A., Mazzarella, L. & Sica, F. Duplex-quadruplex motifs in a peculiar structural organization cooperatively contribute to thrombin binding of a DNA aptamer. *Acta Cryst. D* **69**, 2403-2411 (2013).

6 Horvath, M.P. & Schultz, S.C. DNA G-quartets in a 1.86 angstrom resolution structure of an Oxytricha nova telomeric protein-DNA complex. *J. Mol. Biol.* **310**, 367-377 (2001).

7 Lorenz, R. *et al.* ViennaRNA Package 2.0. *Algorithms for molecular biology : AMB* **6**, 26 (2011).

8 Popenda, M. *et al.* Automated 3D structure composition for large RNAs. *Nucleic Acids Res.* **40**, e112 (2012).
